# Exploring How Patients Are Supported to Use Online Services in Primary Care in England Through “Digital Facilitation”: Survey Study

**DOI:** 10.2196/56528

**Published:** 2024-08-07

**Authors:** Rachel Winder, John L Campbell, Nurunnahar Akter, Abodunrin Q Aminu, Jeffrey Lambert, Emma Cockcroft, Chloe Thomas, Christopher E Clark, Carol Bryce, Jon Sussex, Helen Atherton, Christine Marriott, Gary Abel

**Affiliations:** 1 University of Exeter Medical School University of Exeter Exeter United Kingdom; 2 University of Liverpool Department of Health Data Science Liverpool United Kingdom; 3 Department for Health University of Bath Bath United Kingdom; 4 Unit of Academic Primary Care University of Warwick Warwick United Kingdom; 5 RAND Europe Cambridge United Kingdom; 6 Primary Care Research Centre University of Southampton Southampton United Kingdom

**Keywords:** primary care, online services, access to online health care services, general practice, survey, digital support, inequalities, remote consultation, health services research, digital technology

## Abstract

**Background:**

Health service policy in many jurisdictions is driving greater investment into digital primary care services. While some patients and practices may benefit, there are concerns that not all are able or wish to access primary care services online. “Digital facilitation” is the “range of processes, procedures, and personnel seeking to support patients in their uptake and use of online services” and may address such concerns.

**Objective:**

As part of a multimethod research program, we undertook surveys of practice staff and patients to gain insight into the support being offered by practices and explore patients’ experiences of this support.

**Methods:**

General practices from 4 regions of England were sent a questionnaire exploring the modes of digital facilitation offered, the personnel involved in its delivery, and views on the motivations and drivers for providing support. Moreover, 12,822 patients registered with 62 general practices (predominantly those providing practice survey responses) were sent a questionnaire exploring their experiences of any support offered by their practice to use online services.

**Results:**

Almost one-third of practices (156/500, 31.2%) responded to the practice survey, with most reporting using passive modes of digital facilitation (eg, display, leaflets, and SMS text messages) and few using active modes (eg, offering tablets or computers or using practice champions). However, 90.9% (130/143) reported providing ad hoc support. Practices agreed that it was the responsibility of both the practice (105/144, 72.9%) and the wider National Health Service (118/143, 82.5%) to support patients in using online services and that providing such support benefited the practice (126/144, 87.5%) and their patients (132/144, 91.7%). Nearly a quarter of the patients (3051/12,822, 23.8%) responded to the patient survey, with few (522/3051, 17.11% or less) reporting awareness of any modes of digital facilitation apart from text messages and emails (1205/3051, 39.5%) and only 13.36% (392/2935) reporting receiving support to use online services. Adjusted logistic regression analyses showed that older patients had a lower likelihood of 4 outcomes: being aware of, or of using, digital facilitation efforts, or being told about or being helped to use online services (all *P*<.05), particularly with regard to being helped to use online services (adjusted odds ratio for patients aged 85 years versus those aged 55-64 years: 0.08, 95% CI 0.02-0.36). However, ethnic minority participants or those for whom their first language was not English had positive associations with these outcomes.

**Conclusions:**

General practices recognize that patients would benefit from support to access online services. However, the support provided is often passive or ad hoc, and patients were seldom aware of digital facilitation efforts that their practice provided. There is potential to increase engagement with online primary care services by providing more support for all patients, particularly to provide targeted support for older patients.

## Introduction

### Background

The UK National Health Service (NHS) is experiencing increasing demand for primary care provision as a result of demographic change, technological progress, and changing service configuration [1,2]. In a drive by NHS policy makers for greater investment in technology to help address the increase in demand, the use of online services in primary care in England is being promoted [3]. Primary care services that are provided online include ordering repeat prescriptions, booking appointments, and checking test results, as well as alternatives to face-to-face consultations between practitioners and patients such as video consults or e-consults [4,5]. There is an assumption among policy makers that online service delivery offers benefits for both patients (eg, wider choice of access, flexibility of use, and convenience) and primary care practices (eg, helping to organize work via triage to a specific person or service), thus suggesting the need for promotion or advocacy in respect of the use of such services [6]. The *NHS Long Term Plan*, published in 2019, aimed to ensure that every patient was offered access to digital primary care services and that all general practitioner (GP) practices had a website [7]. The COVID-19 pandemic created the need for urgent action to increase access to, and the use of, online services to reduce infection risk [8,9]. More recently, the 2023 model of general practice care aimed to enable practices to optimize patients’ access to primary care services by all routes, including by online access [2].

Despite benefits for some patients [10,11], there are concerns that not all are able or wish to access their general practice (primary medical care) services online [12,13]. Gaining such access depends on having the technology, the knowhow, and a willingness to use such services [12,14,15]. Some patients may need support to set up their access and then to continue using an online service [16].

“Digital facilitation” refers to “the range of processes, procedures and personnel seeking to support patients in their uptake and use of online services” [16-18]. While there are programs aimed at widening digital participation and providing support for digitally excluded people [19,20], there is little formal evidence to suggest that patients who need support and help to access and use digital services in health care settings are receiving it; furthermore, where it is available, it is unclear what form such support might be taking.

### Surveys of Practice Staff and Patients

The wider mixed methods study [18] aimed to describe the range of digital facilitation activities in general practice in England and included a scoping review [16]; practice and patient surveys; an analysis of national patient survey data; an ethnographic case study, including observations of general practice staff; patient and stakeholder interviews; and a synthesis of the evidence from the study. The surveys of practice staff and patients, as detailed in this paper, aimed to gain insight into what support (if any) to use online primary care services was being offered by practices and to explore patients’ experiences of this support.

## Methods

### Sampling and Distribution

Our practice sampling frame was identified using publicly available information covering 8 NHS commissioning group areas (610 practices) in England. Between December 2020 and May 2021, we approached a random sample of 500 of these practices, initially by the practices’ generic email address or via practice website forms, and included a link in the email to complete the survey online (specific to that practice), with up to 3 reminders to nonresponders at intervals of 2 to 3 weeks. Approximately 8 weeks after initial contact, paper questionnaires with reply envelopes were posted to nonresponding practices for the attention of the practice manager and up to 4 named GPs listed as providing services at the practice. Potential respondents could respond via a survey link to the survey or complete the paper copy and post it back in a reply-paid envelope. Only the first complete response per practice was used in the analysis. We anticipated responses from 300 (60%) of the 500 practices by using this approach [21].

All practices from which we received a response in the practice survey were invited to participate in the patient survey. We sought to invite 12,000 patients aged ≥16 years across 60 practices, aiming to secure 4200 (35%) responses [22]. Practices were given detailed instructions on how to select a random sample of up to 320 eligible patients per practice. Practices were requested to check the list and exclude patients known to be experiencing severe mental illness or recent bereavement, as well as those unable to provide informed consent. The remainder (up to 285 patients per practice) were mailed a paper questionnaire by their practice (along with an invitation letter, information sheet, and a reply-paid envelope). Subsequently, 2 reminders were sent from the practice to all invitees over the next month. The research team did not have access to identifiable information on participating patients, and practices were not aware of details of who had responded. All materials were presented in English (although, for the patient survey, the invitation advised that another person could complete the survey on behalf of the respondent, and a telephone number was provided for help with completion). In line with recognized survey processes [23,24], practices located in areas with populations experiencing relatively high levels of deprivation (according to their Index of Multiple Deprivation score) were requested to invite larger numbers of patients to participate.

The invitation letter and information sheets for each survey explained the purpose of the study; the expected time to complete it (10 minutes for the practice survey and 10-15 minutes for the patient survey); which data would be stored how, where, and for how long; and the names of the investigators.

Any data received via responses on paper were entered by the study team using the same online survey platform. To ensure accurate data entry, double data entry was performed for 4 surveys per practice by a separate member of the study team and checked for consistency.

### Survey Instruments

#### Overview

The questionnaires were each developed by subgroups of the research team (and later by the wider research team), with input and feedback from our patient and public involvement and engagement (PPIE) advisory group, in addition to convenience samples of patients, GPs, and volunteers who provided feedback on usability and functionality. The questions were developed using an iterative process and drew on the findings of the recent scoping review of digital facilitation [16] that formed part of the wider study [18].

#### Practice Survey

The practice survey (Multimedia Appendix 1) consisted of 11 main items presented across 4 sections (“Promotion, help and support for patients to use online services,” “Changes in access to offline services since national lockdown,” “Your views,” and “Your role”). No scales were developed. The items were formulated to address the range of online services offered to patients at the time of the survey and before the COVID-19 pandemic (ie, before 2020), the activities used to promote online services or to support patients in using them, the staff involved in supporting patients, whether specific groups were targeted with this support, respondents’ views on responsibility for support, the influence of various factors relating to online service provision, and the rationale behind providing online services. For the purposes of the questionnaire, promotion activities were defined as “activities to either inform patients about online services or encourage their use without necessarily providing any help or ‘support’ to assist patients in using them.” Response options were generally *tick box* in nature with a 5-point Likert scale for items in the “Your views” section. One question provided the opportunity for an open, free-text response on any issues that the respondent saw as relevant (which will be reported elsewhere). Practice staff could respond using the paper questionnaire they were sent (and return it in the reply-paid envelope) or complete online via an individual link.

#### Patient Survey

The content of the patient survey included items based on existing questionnaires on computer competence and confidence [25-28] and the national General Practice Patient Survey of NHS patients in England [29]. Patient survey questions (Multimedia Appendix 2) reflected the content of the practice survey, with wording tailored for patients. In addition to sections exploring patients’ digital confidence, the survey included sections on patients’ awareness and uptake of online services, as well as their experience of any support provided by the practice to use online services. Two further questions asked respondents about what help the practice could provide to access and use their practice’s online services, the results of which are reported elsewhere [30]. The questionnaire was piloted with a convenience sample of 6 volunteers (members of the public: n=3, 50%; members of the PPIE group: n=3, 50%).

### PPIE Group Input

This project was conducted in collaboration with a study-specific PPIE group, including both patients and caregivers. Eight PPIE group members participated in the development, methods, and interpretation of findings of the practice and patient surveys. The group attended an initial brainstorming session for the patient survey. At further meetings, the PPIE contributors generated discussion on whether or how to include points in the surveys. As a result of the input of the PPIE contributors, the research team added and amended some response options (eg, use of emails to and from practices as a further response choice); reworded some digital facilitation options (eg, included television displays in GP surgeries); incorporated further suggested inclusions (eg those with carer responsibilities as a potential group that practices might target with digital facilitation); explored further which patient groups may have needed targeted support when using online services; and identified words that might cause a barrier to respondents understanding the questionnaire (eg, “activities,” “facilitation,” and “engage with”), which were removed. The contributors also provided feedback on aspects of completing the survey and its length and requested further explanation of acronyms and abbreviations used in the patient invitation letter. The contributors attended meetings where the survey results were presented and discussed and contributed to the interpretation of initial findings from a patient perspective.

### Ethical Considerations

Ethics approval was granted for the patient survey by the North East Newcastle and North Tyneside 2 Research Ethics Committee on April 27, 2021, and by the Health Research Authority on July 1, 2021 (Integrated Research Authority System 289425). Ethics approval was not required for the practice survey element (as advised by the Health Research Authority) because the survey did not intend to change practice or patient care. Patients were deemed to have consented to participate in the patient survey if they returned a questionnaire either by post or online (implied consent). The research team did not ask for any personal data from survey participants, although participants could provide their contact details (which were kept separate from other survey data) if they wished to take part in the prize draw. Information on processing of personal data on the participant information sheet provided an explanation of our approach to handling personal data. Analysis of General Practice Patient Survey data was deemed service evaluation not requiring ethics approval.

Practices responding to the practice survey were entered into a prize draw for 1 of 10 £250 (US $316) vouchers. A voluntary prize draw for 1 of 10 £25 (US $32) vouchers was offered as an incentive for patients participating in the patients survey. Potential patient survey respondents were informed that consent would be assumed upon return of a questionnaire either by post or online.

### Statistical Analysis

#### Examining Variability in Digital Facilitation Outcomes

Simple descriptives were used for the practice and patient surveys (after excluding missing responses to individual questions). The patient survey data were further analyzed using mixed effects logistic regression models, with a random effect for practice, to examine variability in 4 outcomes in relation to patient characteristics on a complete case basis. The four binary outcomes were developed from patient survey responses, considering (1) awareness of, and (2) use of, practice efforts aimed at providing digital facilitation (ie, by endorsing 1 of the following options in the relevant questions [Q10 and Q11]: displays in the practice, leaflets, email or text messages, practice website, social media, workshops or events, and making IT equipment available to access online services); (3) being told by someone from the practice about online services; and (4) being helped by someone from the practice to use online services. For each of the binary outcomes, univariable associations were calculated along with a multivariable model adjusting for respondent self-reported age, gender, deafness or hearing impairment, parental status, ethnicity, physical or mental long-term health condition, working status, first language, and whether they had repeat prescriptions (adjusted model 1). A second model (adjusted model 2) augmented adjusted model 1 with a composite measure of digital confidence constructed from patient responses to Q2 to Q6 (Multimedia Appendix 3). A further regression was run in this model using a binary version of the digital confidence scale (confident vs quite confident or not confident) as the outcome and using the same covariates as in adjusted model 1. Sensitivity analyses were performed, excluding respondents who specified having received help to complete the survey.

#### Comparison of Practice and Patient Survey Response Data: Combined Analyses

Finally, we combined data from the practice and patient surveys (restricted to practices that participated in both parts of the study) to examine whether practice responses were associated with patient awareness and use of digital facilitation. Three sets of comparisons were made using chi-square tests:

- The percentage of respondents reporting being aware of, and the percentage of respondents reporting using, particular modes of facilitation were compared between those registered at practices that reported offering that mode of facilitation and those registered at practices that did not make such a provision (eg, we compared the percentage of respondents who were aware of their practice providing leaflets about online services between practices that said they used leaflets to promote and support the use of online services and those that did not).
- The percentage of respondents reporting being aware of, and the percentage of respondents reporting using, any mode of facilitation were compared: (1) between respondents registered at practices that reported undertaking ad hoc promotion or support of online services and those that did not and (2) between respondents registered at practices that reported using a practice champion and those that did not (eg, we compared the percentage of respondents who were aware of any mode of facilitation between practices that said they used a practice champion and those that did not).
- We investigated respondent groups who described themselves as either an older adult (aged ≥65 years), having a physical health condition, having a mental health condition, having limited or no internet access, being a non-English speaker or speaking English as a second language, being a member of an ethnic minority, or as being a carer. We compared the percentage, separately, in each of these groups, who reported awareness or use of any modes of facilitation between those registered at practices that reported offering digital facilitation support targeted at that group and those that did not (eg, we compared the percentage of respondents aged ≥65 years who were aware of, or used, any mode of facilitation between practices that said they targeted older patients and those that did not).

### Study Registration

The study was registered with the Research Registry (researchregistry6523).

## Results

### Practice Survey

Of the 499 practices invited (of the intended 500 practices, n=2, 0.4% were found to have merged), 156 (31.3%) sent back at least 1 questionnaire. Participating practices were more likely than other practices in England to serve less deprived populations (21/155, 13.6% in the least deprived quintile vs 1359/6745, 20.15%), to be in rural areas (46/156, 29.5% vs 1004/6842, 14.67%), and to have >12,000 registered patients (54/155, 34.8% vs 1477/6461, 22.86% [31-33]; Multimedia Appendix 4). The age profile and ethnicity of the registered populations showed smaller differences compared to other practices. Full summaries of survey responses are presented in Multimedia Appendices 4-12, but here we focus on those questions pertaining to digital facilitation.

**Figure 1:**
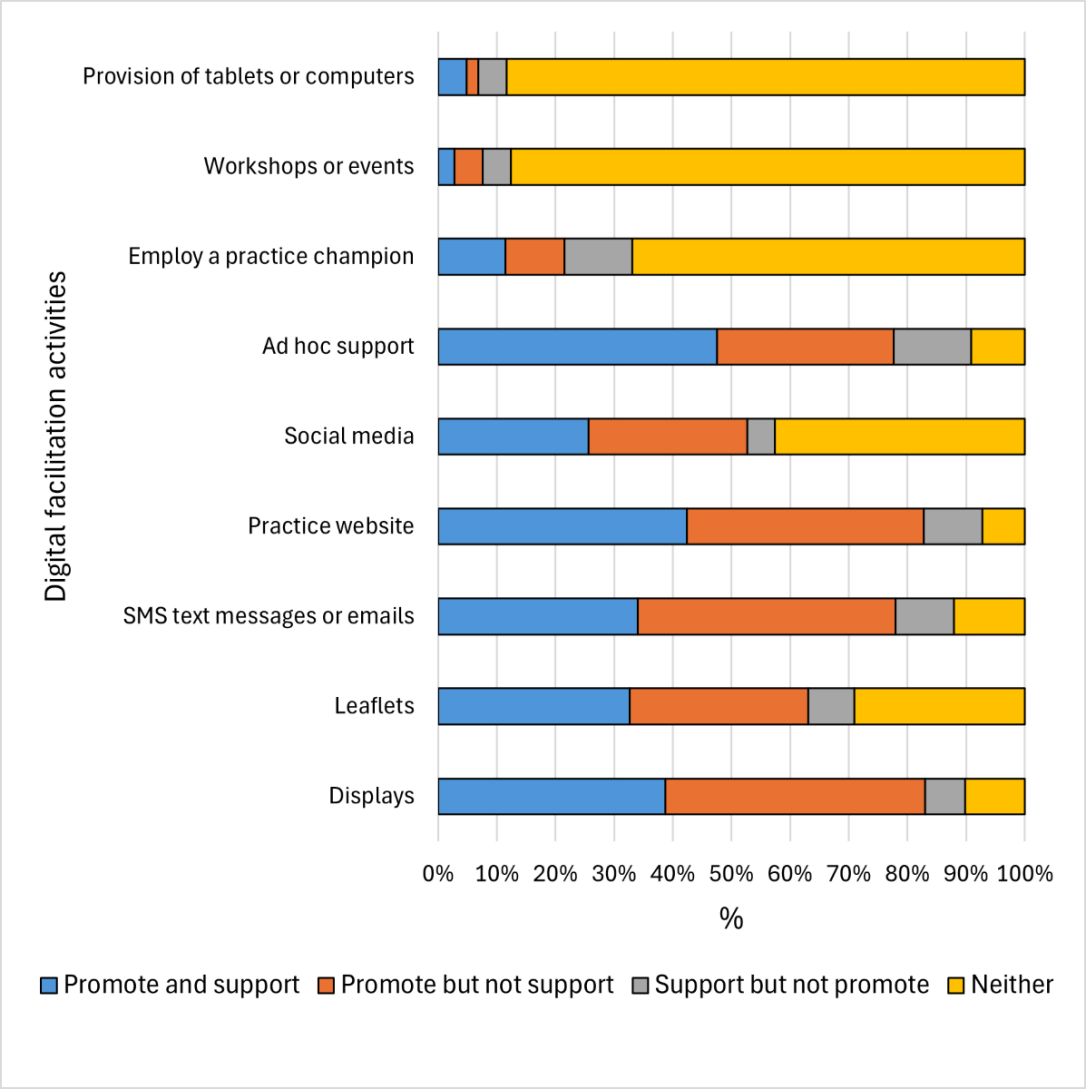
Digital facilitation activities used to promote online services and support patients to use them by percentage of practices responding to the practice survey.

There was a clear division in the practice survey between the endorsement of modes of digital facilitation that could be described as passive, where information is provided with no scope for 2-way interaction (displays, leaflets, text messages, emails, social media, and material on practice websites), and those that could be described as active and would involve 2-way interaction between patients and staff members (ad hoc support, using a practice champion, holding workshops or events, and offering tablets or computers; Multimedia Appendix 6). A majority of practices reported using passive modes of facilitation for either promotion of online services or supporting patients to use them; for example, leaflets were used in 70.9% (100/141) of the practices (Figure 1). By contrast, with the exception of ad hoc support (which was reported in 130/143, 90.9% of the practices), active modes of facilitation were reported by only a minority of practices (eg, workshops or events were used by 18/145, 12.4% of the practices). Most of the practices reported using digital facilitation across a wide range of online services, with 96.5% (139/144) reporting using it to promote or support online repeat prescription ordering (Multimedia Appendix 7).

Of the 156 participating practices, the vast majority reported that promotion and supporting activities involved administrative (n=134, 85.9%) and reception (n=134, 85.9%) staff, with doctors (n=96, 61.5%), nurses (n=83, 53.2%), and other health care professionals (n=65, 41.7%) also frequently reported as being involved. The involvement of IT staff (44/156, 28.2%), volunteers (31/156, 19.9%), external contractors (6/156, 3.9%), and practice staff with specific responsibilities around digital facilitation (16/156, 10.3%) was reported less often (Multimedia Appendix 8). Of the 156 practices, 18 (11.5%) reported that they targeted their facilitation activities at every patient group listed in the questionnaire (Multimedia Appendix 9), with most of the practices (85/156, 54.5%) targeting >1 group. The only specific group reported to be targeted by most of the practices (87/156, 55.8%) were older adults aged ≥65 years (Multimedia Appendix 9).

Most of the practices (129/144, 89.6%) responding to a list of statements (Multimedia Appendix 11) agreed that the COVID-19 pandemic had been a key driver in the uptake of online services by patients, and many (109/144, 75.7%) reported that the pandemic had led to an increase in the support they provided to patients to use online services. Responsibility to alert patients about online services was seen to lie with the practice (123/144, 85.4%) or the wider NHS (122/144, 84.7%), with many also seeing responsibility of providing support to use online services lying with the practice (105/144, 72.9%) and the NHS (118/143, 82.5%). Current support provided to patients by practices was agreed to be in response to patient demand (119/142, 83.8%), rather than the Clinical Commissioning Group (47/142, 33.1%), practice staff (61/142, 43%), or monetary incentives (20/135, 14.8%).

Nevertheless, most of the respondents agreed that supporting patients to use online services benefited the practice (126/144, 87.5%) and patients (132/144, 91.7%); however, 60.3% (85/141) of the practices agreed that the practice lacked the capacity to provide adequate support for patients. Indeed, 78.4% (109/139) agreed that their practice had increased the uptake of online services by supporting patients to use them, and 72.7% (104/143) hoped to further increase uptake through support. It was acknowledged by nearly all respondents (135/143, 94.4%) that some patients were unlikely to use online services regardless of the support provided and that some patient groups required more support than others (131/143, 91.6%). Two-thirds of practice respondents (95/142, 66.9%) agreed that increasing patient uptake of online primary care services led to operational efficiencies for the practice. There was strong agreement that online access to primary care services was complementary to traditional forms of access (121/143, 84.6%), whereas 36.6% (52/142) agreed that online primary care services would ultimately replace traditional forms of access.

### Patient Survey

In all, 62 practices sent invitations to 12,822 patients between August 2021 and May 2022, of whom 3051 (23.8%) responded (Multimedia Appendix 13). Analysis of double data entry of paper responses identified a difference in 0.4% of data points entered, which was considered acceptable. Despite lower patient response rates from practices serving populations considered deprived, due to our adopted approach to sampling, responses were largely representative in terms of deprivation with, for example, 21.4% (653/3051) of the responders registered at practices falling in the lowest quintile of deprivation nationally and a broadly even spread of responders (ranging from 438/3051, 14.36% to 698/3051, 22.88%) across all quintiles (Multimedia Appendix 14).

Of those responding to the patient survey, 56.59% (1710/3022) were female, 45.39% (1373/3025) were aged ≥65 years, most (2741/2958, 92.66%) were White, 8.8% (261/2966) reported that English was not their first language, 43.7% (1269/2904) were working either full time or part time, and 42.84% (1244/2904) were retired (Table 1). Of the respondents, 9% (280/3051) said they did not have access to the internet at home, with 98.6% (276/280) of this group completing the paper version of the survey. In terms of overall digital confidence (Multimedia Appendix 3) with respect to a range of digital tasks (eg, using search engines, completing online forms, sending personal messages, and installing apps), a little more than half (1589/2961, 53.66%) were categorized as being very confident, whereas 22.56% (668/2961) were categorized as not confident (Multimedia Appendix 1).

**Table 1:** Self-reported demographics of responders to the main patient survey (n=3051).

Demographic variables		Respondents, n (%)
**Gender (n=3022)**		
	Male	1312 (43.41)
	Female	1710 (56.59)
**Age group (y; n=3025)**		
	16-24	140 (4.63)
	25-34	195 (6.45)
	35-44	262 (8.66)
	45-54	436 (14.41)
	55-64	619 (20.46)
	65-74	727 (24.03)
	75-84	515 (17.02)
	≥85	131 (4.33)
**Deaf or hearing impediment (n=2989)**		
	Yes	358 (11.98)
	No	2631 (88.02)
**Blind or partially sighted (n=2963)**		
	Yes	54 (1.82)
	No	2909 (98.18)
**Ethnicity (n=2958)**		
	Asian or Asian British	107 (3.62)
	Black or African or Caribbean or Black British	57 (1.93)
	White	2741 (92.66)
	Mixed	31 (1.05)
	Other	22 (0.74)
**Mental health condition or disability (n=2876)**		
	Yes	472 (16.41)
	No	2281 (79.31)
	Don’t know	72 (2.50)
	Prefer not to say	51 (1.77)
**Working status (n=2904)**		
	Full-time paid work	906 (31.2)
	Part-time paid work	363 (12.5)
	Full-time student	75 (2.58)
	Unemployed	59 (2.03)
	Permanently sick	72 (2.48)
	Fully retired	1244 (42.84)
	Looking after family	104 (3.58)
	Doing something else	75 (2.58)
	Furloughed^a^	6 (0.21)
**English as their first language (n=2966)**		
	Yes	2705 (91.2)
	No	261 (8.80)
**Carer (n=2937)**		
	Yes	700 (23.83)
	No	2237 (76.17)
**Parent (n=2956)**		
	Yes	420 (14.21)
	No	2536 (85.79)
**Repeat prescriptions (n=2978)**		
	Yes	1957 (65.72)
	No	1021 (34.28)
**Help to complete the survey (n=2963)**		
	Yes	183 (6.18)
	No	2780 (93.82)
**Physical health condition or disabilities (n=2900)**		
	Yes	1046 (36.07)
	No	1737 (59.9)
	Don’t know	74 (2.55)
	Prefer not to say	43 (1.48)

^a^UK government scheme offering payment to employers to retain and pay staff while businesses were closed due to the COVID-19 pandemic.

Table 2 presents a summary of responses to questions concerning awareness, use, and provision of support for the use of general practices’ online services. More than a third of those responding (1106/2998, 36.89%) had not attempted to use their practice’s website, although of those who had tried, 77.27% (1462/1892) reported finding it *very easy* or *fairly easy* to use. For all services considered, ≤37% (≤1126/3051) of the respondents were aware of the various online services provided by their practice, with the exception of appointment booking and ordering repeat prescriptions online (1675/3051, 54.9% and 1944/3051, 63.72%, respectively). In terms of the use of online services, ≤16% (≤501/3051) of the respondents had used a range of online services, with the exception of ordering repeat prescriptions online, where a third (1003/3051, 32.87%) had used the service. Apart from the use of SMS text messages or emails (where 1205/3051, 39.5% respondents reported awareness of facilitation efforts), ≤17% (≤522/3051) of the respondents were alert to any forms of digital facilitation opportunities at their practice. The reported use of these facilitation efforts was even lower than patients’ awareness, with <10% (≤291/3051) of the patients making use of any mode, apart from email or text messages (860/3051, 28.19%).

**Table 2:** Summary of responses to the core items of the patient survey (n=3051).

Questions				Respondents, n (%)
**Ease of practice website use (n=2998)**				
	Very easy			541 (18.05)
	Fairly easy			920 (30.69)
	Not very easy			286 (9.54)
	Not at all easy			145 (4.84)
	Haven’t tried			1106 (36.89)
**Awareness of online services**				
	Appointment booking online			1675 (54.9)
	Ordering repeat prescriptions			1944 (63.72)
	Accessing medical records online			945 (30.97)
	Accessing test results online			663 (21.73)
	Email enquiries to the practice			1126 (36.91)
	Help or advice using an online form (online consultations or e-consults)			945 (30.97)
	Video consultations			458 (15.01)
**Use of online services**				
	Appointment booking online			469 (15.37)
	Ordering repeat prescriptions			1003 (32.87)
	Accessing medical records online			393 (12.88)
	Accessing test results online			260 (8.52)
	Email enquiries to the practice			457 (14.98)
	Help or advice using an online form (online consultations or e-consults)			501 (16.42)
	Video consultations			118 (3.87)
**Awareness of facilitation**				
	Displays in the practice (eg, posters or television displays)			522 (17.11)
	Leaflets about online services			178 (5.83)
	SMS text messages or emails			1205 (39.5)
	Practice website content (eg, *how to* guide or video or prominent pop up)			229 (7.51)
	Use of social media			118 (3.87)
	Scheduled workshop or events (in person or online)			17 (0.56)
	Making tablets or computers available to access online services			16 (0.52)
**Use of facilitation**				
	Displays in the practice (eg, posters or television displays)			291 (9.54)
	Leaflets about online services			93 (3.05)
	SMS text messages or emails			860 (28.19)
	Practice website content (eg, *how to* guide or video or prominent pop-up)			152 (4.98)
	Use of social media			79 (2.59)
	Scheduled workshop or events (in person or online)			15 (0.49)
	Making tablets or computers available to access online services			15 (0.49)
**Usefulness of help**				
	**Booking appointments online (n=196)**			
		Not helpful	40 (20.41)	
		Quite helpful	77 (39.29)	
		Very helpful	79 (40.31)	
	**Ordering repeat prescriptions online (n=211)**			
		Not helpful	22 (10.43)	
		Quite helpful	66 (31.28)	
		Very helpful	123 (58.29)	
	**Accessing medical records online (n=121)**			
		Not helpful	24 (19.83)	
		Quite helpful	42 (34.71)	
		Very helpful	55 (45.45)	
	**Accessing test results online (n=111)**			
		Not helpful	23 (20.72)	
		Quite helpful	34 (30.63)	
		Very helpful	54 (48.65)	
	**Email enquiries to the practice (n=154)**			
		Not helpful	27 (17.53)	
		Quite helpful	53 (34.42)	
		Very helpful	74 (48.05)	
	**Online video consultations with GP** ^a^ **or other health care professional (n=81)**			
		Not helpful	28 (34.57)	
		Quite helpful	19 (23.46)	
		Very helpful	34 (41.98)	
	**Other (n=33)**			
		Not helpful	10 (30.3)	
		Quite helpful	8 (24.24)	
		Very helpful	15 (45.45)	
**Help from GP using online form** **(n=171)**				
	Not helpful			28 (16.37)
	Quite helpful			59 (34.5)
	Very helpful			84 (49.12)
**Told about online services (n=2964)**				
	Yes			883 (29.79)
	No			2081 (70.21)
**Helped to use online services (n=2935)**				
	Yes			392 (13.36)
	No			2543 (86.64)
**Reasons why not using online services**				
	No internet access			247 (8.1)
	Security concern			228 (7.47)
	Confidentiality concerns			145 (4.75)
	Not knowing how to register			440 (14.42)
	Registration too difficult			175 (5.74)
	Not knowing how to get support			259 (8.49)
	Practice too busy to help			262 (8.59)
	Prefer to speak in person			1432 (46.94)

^a^GP: general practitioner.

Only 883 (29.79%) of 2964 patients agreed that they had been told about online services by someone at their practice, and only 392 (13.36%) of 2935 patients reported that they had been supported to use such services. Those who did receive help largely rated this support as beneficial: 89.6% (189/211) selected *quite helpful* or *very helpful* for online ordering of repeat prescriptions, and 65% (53/81) reported the help to be *quite helpful* or *very helpful* for video consultations. A variety of reasons were reported for not using online services, but, most commonly, respondents preferred to speak in person (1432/3051, 46.94%), with 14.42% (440/3051) reporting that they did not know how to register and 5.74% (175/3051) reporting that they found registration too difficult.

Table 3 shows the results of the adjusted model 1 logistic regression analyses, with the results of the unadjusted model analyses shown in Multimedia Appendix 16, for 4 facilitation outcomes, that is, awareness (Q8) and use (Q9) of facilitation efforts, being told about online services (Q12), and being helped to use online services (Q13; Multimedia Appendix 2). Older patients were less likely than younger patients to report awareness and use of facilitation efforts, as well as to report having been told about or helped in the use of such services (all *P* values <.05). This was particularly noticeable in respect of being helped to use online services (adjusted odds ratio [OR] for patients aged ≥85 years vs those aged 55-64 years: 0.08, 95% CI 0.02-0.36; *P*=.006). Respondents in receipt of repeat prescriptions were more likely than those not receiving this provision to have experienced all 4 facilitation outcomes. Patients describing themselves as being of minority ethnicity were more likely than those describing themselves as being of White ethnicity to be aware of digital facilitation (adjusted OR 1.48, 95% CI 1.00-2.20; *P*=.05), to have made use of digital facilitation (adjusted OR 1.48, 95% CI 1.03-2.15; *P*=.04), and to have been helped to use online services (adjusted OR 1.80, 95% CI 1.14-2.86; *P*=.01). However, no difference was seen between respondents of White and minority ethnicity for being told about online services (adjusted OR 0.91, 95% CI 0.61-1.36; *P*=.65). There was weak evidence that patients for whom English was not their first language were more likely to be told about online services (adjusted OR 1.61, 95% CI 1.12-2.32; *P*=.01) or to use digital facilitation (adjusted OR 1.79, 95% CI 1.28-2.52; *P*=.001). The respondent’s gender, being deaf or having a hearing impairment, and parental and employment status were not associated with any of the 4 outcomes (*P*>.10 for all).

**Table 3:** Adjusted logistic regression (model 1) considering participants’ awareness of digital facilitation, use of digital facilitation, being told about online services, and being helped to use online services (all models; n=2587).

Characteristics		Awareness of any digital facilitation efforts^a^		Use of any digital facilitation efforts^b^		Being told about online services		Being helped to use online services	
		Adjusted OR^c^ (95% CI)	*P* value	Adjusted OR (95% CI)	*P* value	Adjusted OR (95% CI)	*P* value	Adjusted OR (95% CI)	*P* value
**Gender**									
	Male	Reference	Reference	Reference	Reference	Reference	Reference	Reference	Reference
	Female	0.92 (0.78-0.90)	.34	0.90 (0.76-1.07)	.23	1.14 (0.95-1.36)	.16	0.86 (0.68-1.10)	.24
**Age group (y)**									
	16-24	0.84 (0.50-1.42)	<.001	0.64 (0.37-1.09)	<.001	1.58 (0.93-2.70)	.04	1.00 (0.47-2.17)	.006
	25-34	1.08 (0.73-1.58)	<.001	1.07 (0.74-1.56)	<.001	1.14 (0.77-1.69)	.04	1.11 (0.65-1.91)	.006
	35-44	0.98 (0.67-1.43)	<.001	1.01 (0.70-1.46)	<.001	1.08 (0.73-1.60)	.04	1.42 (0.85-2.37)	.006
	45-54	0.94 (0.70-1.27)	<.001	0.94 (0.70-1.26)	<.001	1.01 (0.74-1.38)	.04	1.31 (0.87-1.98)	.006
	55-64	Reference	Reference	Reference	Reference	Reference	Reference	Reference	Reference
	65-74	0.71 (0.53-0.95)	<.001	0.88 (0.66-1.17)	<.001	0.77 (0.56-1.05)	.04	0.81 (0.52-1.24)	.006
	75-84	0.43 (0.30-0.60)	<.001	0.62 (0.44-0.87)	<.001	0.66 (0.45-0.95)	.04	0.50 (0.30-0.84)	.006
	≥85	0.32 (0.19-0.54)	<.001	0.27 (0.15-0.49)	<.001	0.38 (0.20-0.71)	.04	0.08 (0.02-0.36)	.006
**Deafness or hearing impairment**									
	No	Reference	Reference	Reference	Reference	Reference	Reference	Reference	Reference
	Yes	0.93 (0.70-1.22)	.60	1.16 (0.87-1.53)	.31	0.82 (0.60-1.13)	.23	1.32 (0.88-2.00)	.18
**Parent**									
	No	Reference	Reference	Reference	Reference	Reference	Reference	Reference	Reference
	Yes	1.28 (0.96-1.72)	.01	1.03 (0.78-1.37)	.83	1.28 (0.95-1.73)	.11	1.03 (0.70-1.52)	.88
**Ethnicity**									
	White	Reference	Reference	Reference	Reference	Reference	Reference	Reference	Reference
	Other	1.48 (1.00-2.20)	.05	1.48 (1.03-2.15)	.04	0.91 (0.61-1.36)	.65	1.80 (1.14-2.86)	.01
**Long-term physical or mental health condition**									
	No	Reference	Reference	Reference	Reference	Reference	Reference	Reference	Reference
	Yes	1.25 (1.03-1.50)	.02	1.17 (0.97-1.41)	.09	1.07 (0.88-1.31)	.48	1.29 (0.98-1.70)	.07
**Work status**									
	Working	Reference	Reference	Reference	Reference	Reference	Reference	Reference	Reference
	Studying	0.73 (0.38-1.41)	.12	1.05 (0.54-2.04)	.19	1.09 (0.56-2.12)	.98	1.23 (0.49-3.10)	.62
	Other	0.72 (0.54-0.95)	.12	0.78 (0.59-1.04)	.19	0.95 (0.70-1.28)	.98	1.23 (0.83-1.82)	.62
	Retired	0.96 (0.72-1.26)	.12	0.77 (0.59-1.02)	.19	1.00 (0.74-1.35)	.98	1.24 (0.82-1.89)	.62
**First language**									
	English	Reference	Reference	Reference	Reference	Reference	Reference	Reference	Reference
	Other	1.38 (0.96-1.96)	.08	1.79 (1.28-2.52)	.001	1.61 (1.12-2.32)	.01	1.52 (0.97-2.37)	.07
**Repeat prescription**									
	No	Reference	Reference	Reference	Reference	Reference	Reference	Reference	Reference
	Yes	1.28 (1.04-1.56)	.02	1.46 (1.19-1.79)	<.001	1.70 (1.36-2.13)	<.001	1.45 (1.06-1.97)	.02

^a^Awareness of any facilitation efforts includes respondents who ticked any of the first 7 options of Q10.

^b^Use of any facilitation efforts includes respondents who ticked any of the first 7 options of Q11.

^c^OR: odds ratio.

In adjusted model 2 (Multimedia Appendix 16), lower digital confidence was associated with both lower awareness and lower use of facilitation, as well as with less likelihood of being told about, or being helped to use, online services. Furthermore, the association between age and each of the outcomes was weaker in this model than in adjusted model 1, indicating that some of the differences ascribed to older age could be explained by older patients having, on average, lower digital confidence than younger patients. However, the changes in ORs were small (eg, OR for the awareness of facilitation for patients aged ≥85 years changed from 0.35 to 0.59), with a substantial age effect remaining after adjustment for digital confidence. By contrast, the association between whether a respondent speaks English as a first language and 3 of the 4 outcomes was stronger when adjusting for digital confidence (eg, OR for being helped to use online services increased from 1.52 to 1.71).

Sensitivity analyses excluding those who reported having had help to complete the questionnaire did not lead to materially different findings (Multimedia Appendix 17).

### Comparison of Practice and Patient Survey Response Data: Combined Analyses

Patients often reported awareness and even use of various modes of facilitation in practices that did not report using that mode. This finding implies that either practice or patient responses were in error or that the practice’s use of particular modes had changed over time. Moreover, when considering specific modes of facilitation, there was generally no evidence that patients registered at practices using that mode were any more likely to report being aware of it or using it; for example, 5.39% (92/1707) of the patients were aware of leaflets being used for digital facilitation in practices that reported using leaflets compared to 6.27% (67/1069) of the patients in practices that did not report using leaflets (Multimedia Appendix 18). However, exceptions were evident for in-practice displays, where 17.55% (466/2655) of the respondents reported awareness of such displays in practices that reported using them compared to 10.7% (30/280) in those that did not (*P=*.004), social media (105/1733, 6.06% vs 12/1242, 0.97% for awareness and 63/1733, 3.64% vs 15/1242, 1.21% for use; *P*<.001 for both), and workshops or events (4/267, 1.5% vs 12/2607, 0.46% for awareness; *P=*.03; and 4/267, 1.5% vs 10/2607, 0.38% for use; *P*=.01).

No difference was evident in respect of patient awareness or use of any form of digital facilitation when comparing those practices that reported using ad hoc support for digital facilitation to those that did not do so (Multimedia Appendix 19). However, while using a practice champion was associated with higher patient awareness of facilitation efforts (149/410, 36.3% vs 669/2304, 29.04%; *P*=.003), no greater use of facilitation efforts was seen (Multimedia Appendix 19). When considering practices that reported targeting specific patient groups with digital facilitation efforts, we found no evidence of differences in the patient awareness or use of any mode of digital facilitation within such groups (Multimedia Appendix 20).

## Discussion

### Principal Findings

We explored whether and how practices supported patients to use online primary care health services and patients’ experiences of this support.

Against a backdrop of practices offering an increased number and variety of online services, in part as a response to the COVID-19 pandemic, our findings showed that practices commonly reported using passive modes (eg, displays, leaflets, and text messages) of digital facilitation to promote or support patients’ use of online services, and reported that practice staff provided ad hoc support for patients. Active modes of digital facilitation (eg, using a practice champion) were rarely in place. We found that administrative (134/156, 85.9%) and reception (134/156, 85.9%) staff provided digital facilitation efforts in the vast majority of practices, with clinical staff (doctors: 96/156, 61.5% and nurses: 83/156, 53.2%) contributing in more than half of the practices. Although most practices viewed the potential for digital facilitation positively, benefitting both patients (132/144, 91.7%) and practices (126/144, 87.5%), many (85/141, 60.3%) felt that they lacked the resources to deliver it.

More than three-quarters of those patients (1461/1892, 77.22%) responding that they had accessed the practice website reported finding it *very easy* or *fairly easy* to use, which is surprising considering that many websites have historically pitched their usability levels too high for many potential users [34]. Nevertheless, despite the relatively positive reports from practices that they have provided some forms of digital facilitation (albeit mainly ad hoc), patients’ reported awareness and use of online services was still generally low. Awareness and provision of any support for the use of these services was lower still, with the exception of text messages or emails, for which 39.5% (1205/3051) of the patients reported awareness of their practice providing support through this mode. Despite older patients tending to have higher health care use and practices reporting that they targeted the group of older adults aged >65 years more often than other patient groups, older patients were less likely to be aware of digital facilitation efforts reported to be provided by their practice or to use them or even to report having been informed about online services or being supported in their potential use of such services.

### Comparison to Prior Work

Recent reports have focused on inequalities in accessing health services within the NHS from ethnic minority and other groups [35,36]. Perhaps surprisingly, in our study, ethnic minority individuals and those for whom English was not their first language were more likely to report engagement with, or being engaged in, digital facilitation. Being in receipt of repeat prescriptions was also associated with such engagement. Patients gave a variety of reasons for not engaging with online services, often expressing their preference to speak to someone and their difficulties in registering to use online services. Such difficulties suggest unmet need for effective digital facilitation to increase the use of online services.

It is not surprising that when people are not aware of, and do not use, online services, their awareness of any support to use them would be lower still. However, it is important to consider whether a lack of support leads to a reduced use of online services. Low patient awareness and use of online services has been shown in other surveys of patients. In a survey of general practice patients in the West Midlands of England in 2019, half (1362/2715, 50.17%) were aware they could order repeat prescriptions online, and 21.84% (584/2674) had used the service; for accessing records, 23.17% (629/2715) were aware, and 7.79% (172/2207) had used them [37]. Our study showed some increase on these figures but with levels remaining low overall. The same survey found that patients who used the internet daily were more likely to be aware of, and use, online services, suggesting that familiarity with the internet leads to the use of online services, and this is aligned with our findings that the level of digital confidence is associated with awareness and use of online services [37]. Ordering repeat prescriptions would, for many, mean frequent use of the online repeat prescription service. This higher engagement, evident in patients in our study, may have helped them embed the online process, or necessitated the need for them to seek out support.

Research applying secondary analysis of the English General Practice Patient Survey to examine awareness and use of online appointment booking reported that 45% of patients were aware of it, and 16% had used it; these values are comparable with our findings [38]. Qualitative research in the same study identified that older patients preferred other ways of booking appointments and that difficulties registering for online appointment booking impacted their decision on whether to use it [38]. In our study, 5.74% (175/3051) of the respondents reported registration being too difficult, and 14.42% (440/3051) reported not knowing how to register, as the reasons why they did not use online services.

A 2016 survey reporting on how UK general practices were providing (or planning) digital consultations (email and video) [21] found that GPs were concerned about the impact that digital consultation could have on older people. Our survey of practices found that digital facilitation efforts were mainly targeted at older adults, which fits with the perception that it is older adults who struggle most with digital health services. For the older population, Leach et al [16] found that despite the concern that older people need additional support, there were some studies that showed that older people were more likely to use technology to access online services once provided with digital facilitation efforts [39-41]. In considering ethnicity and language, despite reports on the implications of language barriers and their effect on health [42,43], there is a need to explore further whether online services are offering improved access via online over face-to-face services. It is possible that those of minority ethnicity and those whose first language was not English were more used to finding technological workarounds or have family and friends who can support them to use the services.

A Norwegian study examining older patients’ use of e-consults found that they had neither been encouraged to use the service nor helped to use it, and patients highlighted the importance of having digital literacy to use the service [44]. Previous research examining the unintended consequences of introducing digital services in general practice found that patients expressed uncertainty about how to use online consultation systems and their online patient records, and that this could be countered by providing patients with clear information and instructions on how to use them [14,45]. In 1 study, there was some evidence that the more proactive, *hands-on* facilitation approaches were likely to be of more use and support for patients from older age groups [16].

### Strengths and Limitations

One of the strengths of the study was that by oversampling patients in deprived areas, we gained a broadly similar number of responses from patients across all deprivation quintiles. The survey procedures were refined by conducting a pilot survey and was supported by extensive patient and public input in the design and implementation of the research. This work was undertaken as part of a large-scale research project that got underway as the COVID-19 pandemic broke out, which presented many challenges. However, we were able to deliver the surveys and provided added insights into a system of access to practices that was changing rapidly.

Second, our patient survey response rate (3051/12,822, 23.8%) is comparable to that of other primary care–based surveys [37,46,47] and should be considered credible when bearing in mind the difficulties also presented to patients by the COVID-19 pandemic. We also managed to capture many responses from those respondents reporting no access to the internet by using paper invites as well as offering an online mode. Of the 280 respondents reporting no internet access, 276 (98.6%) took advantage of the paper version of the survey. At the start of the pandemic, practices were grappling with new and rapidly changing procedures, increasing staff absenteeism, and increased service use [48,49], and thus participation in research for some practices could have been too challenging. The moderate response rate for the patient survey could also have been a product of our wish to target patients in more deprived areas.

Third, by focusing the patient survey on practices that had responded to the practice survey, we were able to link the responses from the 2 surveys. However, often, the types of facilitation reported by practices had no statistical association with patient responses. This may be a limitation of using self-report, with responses being unreliable, or an indication that the intensity of digital facilitation efforts was too low to impact many patients. This uncertainty could not be investigated in detail because we were not able to examine the intensity of facilitation efforts.

Considering limitations, first, all materials for the patient survey were presented in English. Nevertheless, 8.8% (261/2966) of the respondents reported that their first language was not English, which is similar to the percentage reported in the UK Census 2021 for England and Wales (8.9%) [50]. Our advice that another person could complete the survey from the respondent’s viewpoint on their behalf may have encouraged some who felt that they could not complete it without help.

A further limitation to the study was that we did not have information on the socioeconomic status (eg, deprivation) of patients or the rurality classification of their home location. We were not privy to patient addresses, which would allow linkage to data sets containing this information.

### Future Directions

Despite the high levels of ad hoc support reported to be provided by practice staff in our survey, only 13.36% (392/2935) of the respondents to the patient survey reported having been supported to use an online service. It is possible that the lack of any formality in the process of receiving support was not recognized as bona fide assistance in their online endeavors, or it may be that most of the ad hoc support is provided to a small proportion of a practice’s patients. Establishing whether this is truly the case should be a focus for future research.

Our findings raise questions regarding the success of NHS policy to increase patients’ reliance on online services [51]. They draw further attention to the need for policy makers to provide resources in the form of finances for staff training, time, and infrastructure for practices to ensure that all patients have equitable access to online primary care services, as well as to allow for the ongoing increasing use of online services by patients [18,40,52-54].

### Conclusions

There is a range of digital facilitation activities offered at general practices. This is limited to passive or ad hoc forms in most general practices, with few providing more proactive models of support. There is a recognition by general practices that patients would benefit from support in accessing and using online services. Despite most practices reporting provision of digital facilitation efforts, most patients are not aware of them. However, where patients do experience these facilitation efforts, they often find them helpful. Some patient groups are at risk of digital exclusion [15,20], and it is potentially concerning that older patients are less likely to be aware of, or make use of, digital facilitation efforts. More reassuringly, ethnic minority patients and those for whom English is not their first language seem to be better targeted. Given the number of patients who do not know how to access online services or do not know how to get support in using them, there is unmet need for increasing engagement with online primary care services by providing more digital facilitation to support the rollout of digital services and particularly to provide targeted support for older patients.

## Appendix

Multimedia Appendix 1 Practice survey: questionnaire.

Multimedia Appendix 2 Patient survey: questionnaire.

Multimedia Appendix 3 Digital confidence score development.

Multimedia Appendix 4 Patients in practices in England: comparisons between survey practices and other practices.

Multimedia Appendix 5 Online services offered by practices: current and before the COVID-19 pandemic.

Multimedia Appendix 6 Practice activities to promote, help or support patients use online services.

Multimedia Appendix 7 Services that have been promoted and supported by practices.

Multimedia Appendix 8 Staff roles involved.

Multimedia Appendix 9 Practice responses to questions about patient groups.

Multimedia Appendix 10 Removal or reduction of offline services since the start of the COVID-19 pandemic.

Multimedia Appendix 11 Practice survey: agreement or disagreement with statements.

Multimedia Appendix 12 Awareness of online toolkits.

Multimedia Appendix 13 Practice recruitment, patient survey invitation, and response.

Multimedia Appendix 14 Patient survey: invitations and response by deprivation quintile.

Multimedia Appendix 15 Patient survey: digital confidence score.

Multimedia Appendix 16 Logistic regression analyses: unadjusted model 1 and adjusted model 2.

Multimedia Appendix 17 Sensitivity analyses: adjusted models 1 and 2 (excludes those responding yes to the question “Did you need help to complete this survey?”).

Multimedia Appendix 18 Comparison of patient-reported awareness and the use of modes of digital facilitation with practice-reported use of facilitation mode.

Multimedia Appendix 19 Combined analyses of practice and patient surveys: patient awareness.

Multimedia Appendix 20 Combined analyses of practice and patient surveys: targeted patient awareness.

## Abbreviations

GP: general practitioner
NHS: National Health Service
OR: odds ratio
PPIE: patient and public involvement and engagement
