# Supplementary figures and images for: Exploring How Patients Are Supported to Use Online Services in Primary Care in England Through “Digital Facilitation”: Survey Study
Source: J Med Internet Res. 2024 Aug 7;26:e56528. doi: 10.2196/56528 (PMC11339568; doi:10.2196/56528)

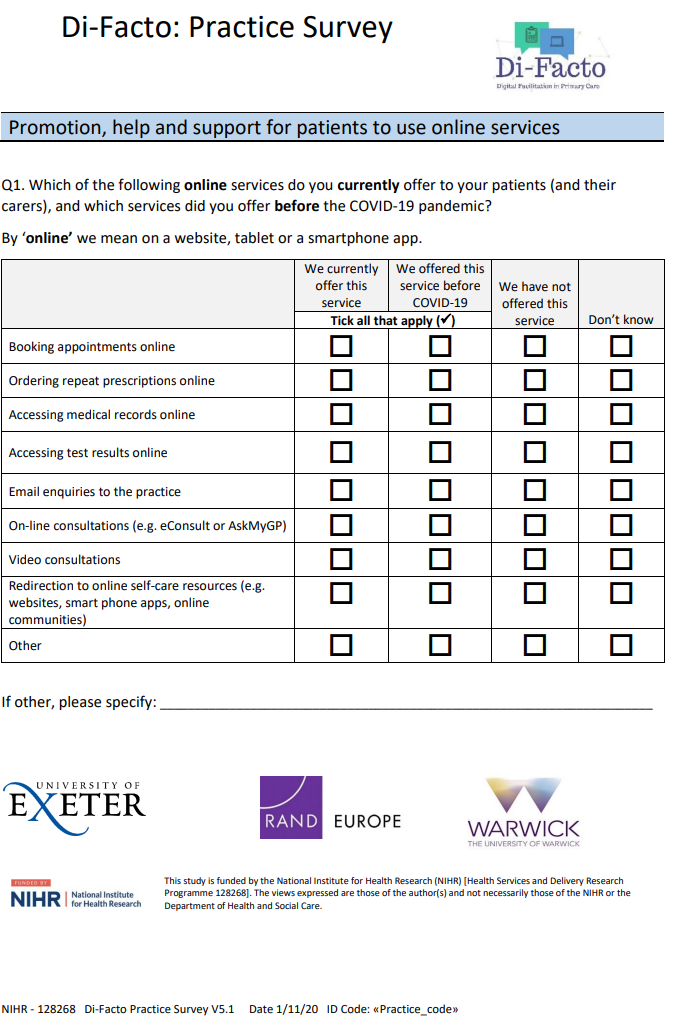


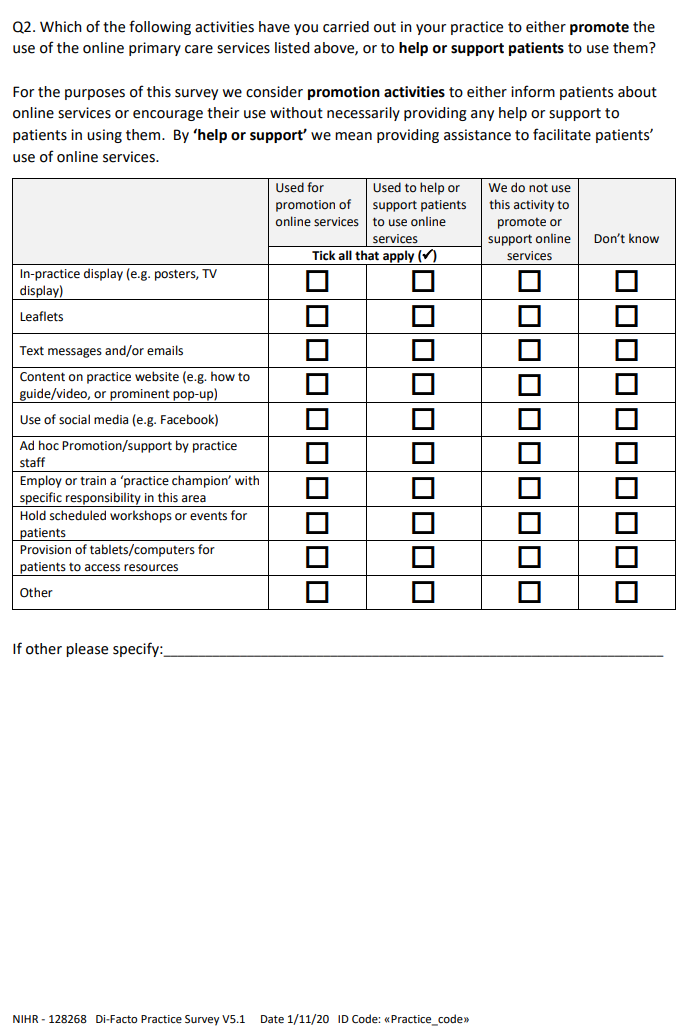


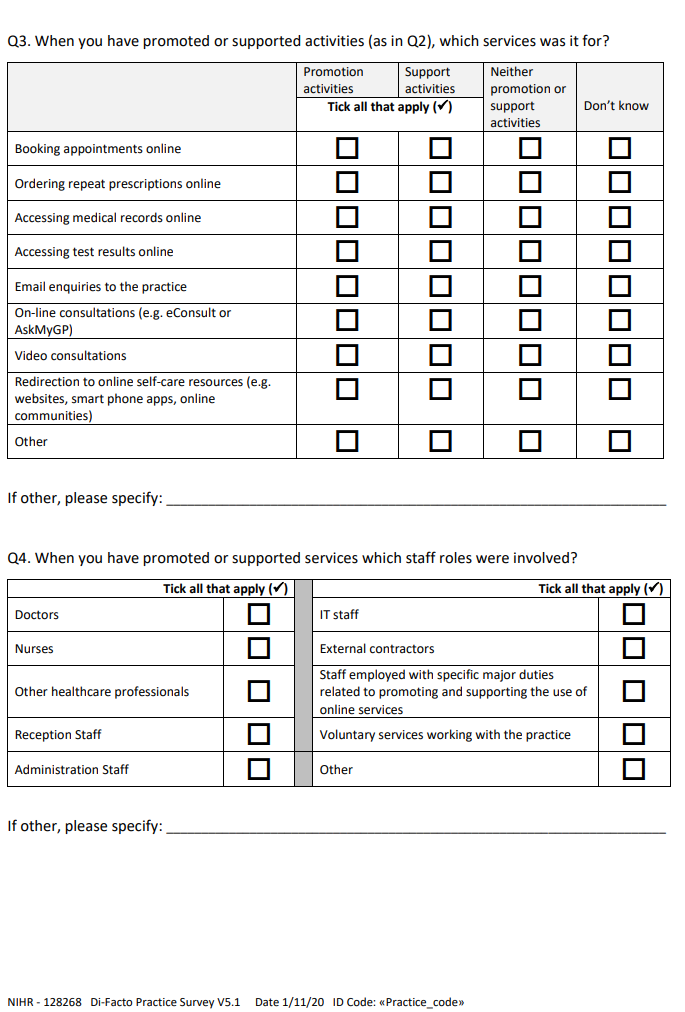


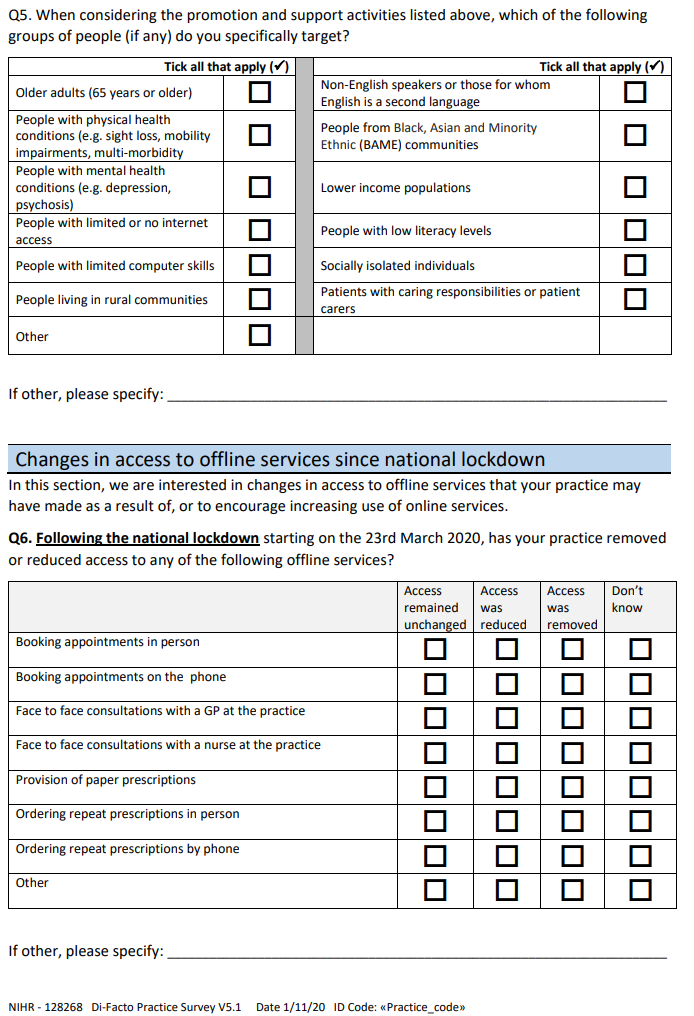


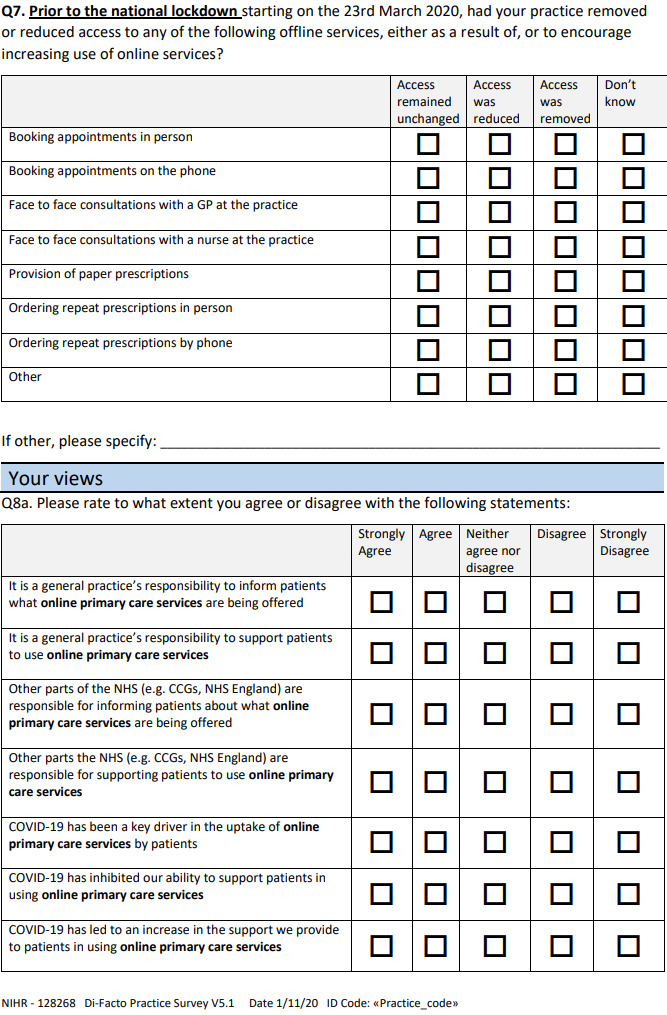


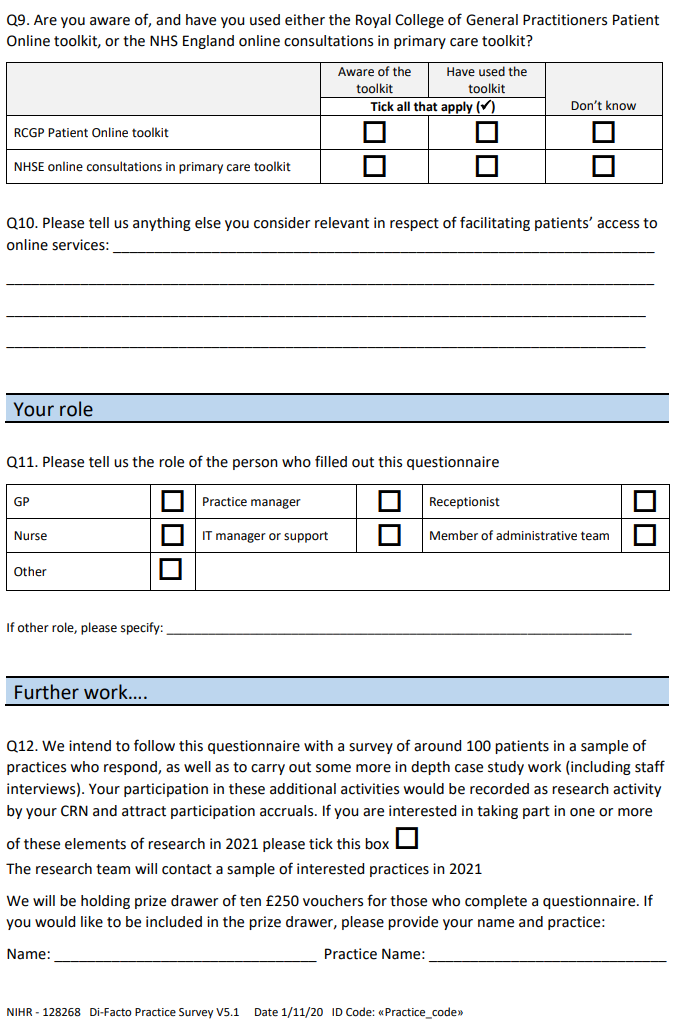

Supplement: Multimedia Appendix 1 [file jmir_v26i1e56528_app1.docx]

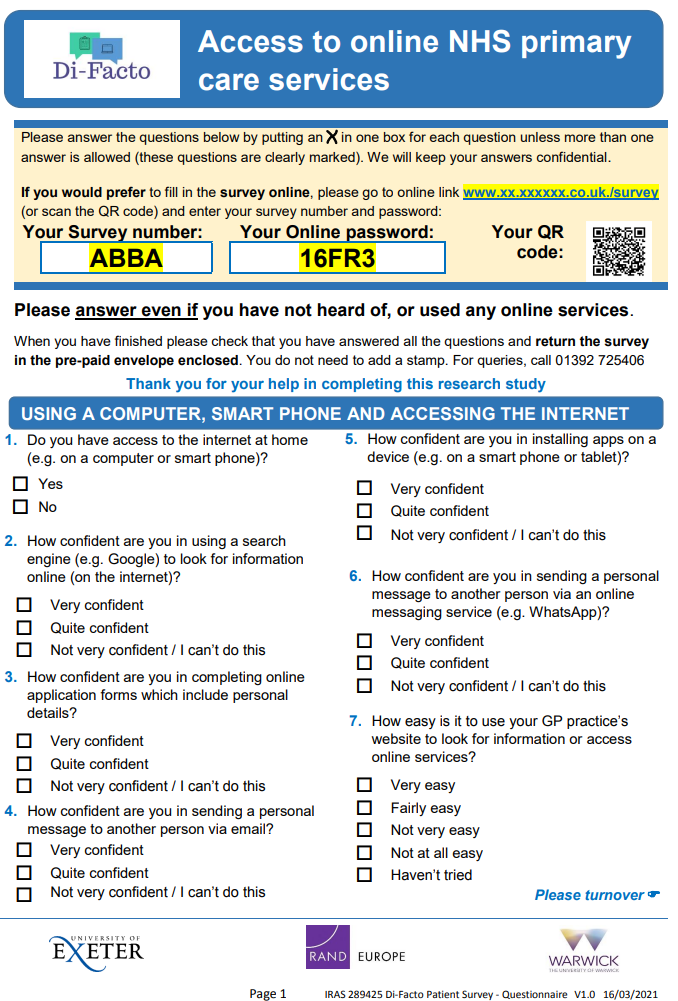


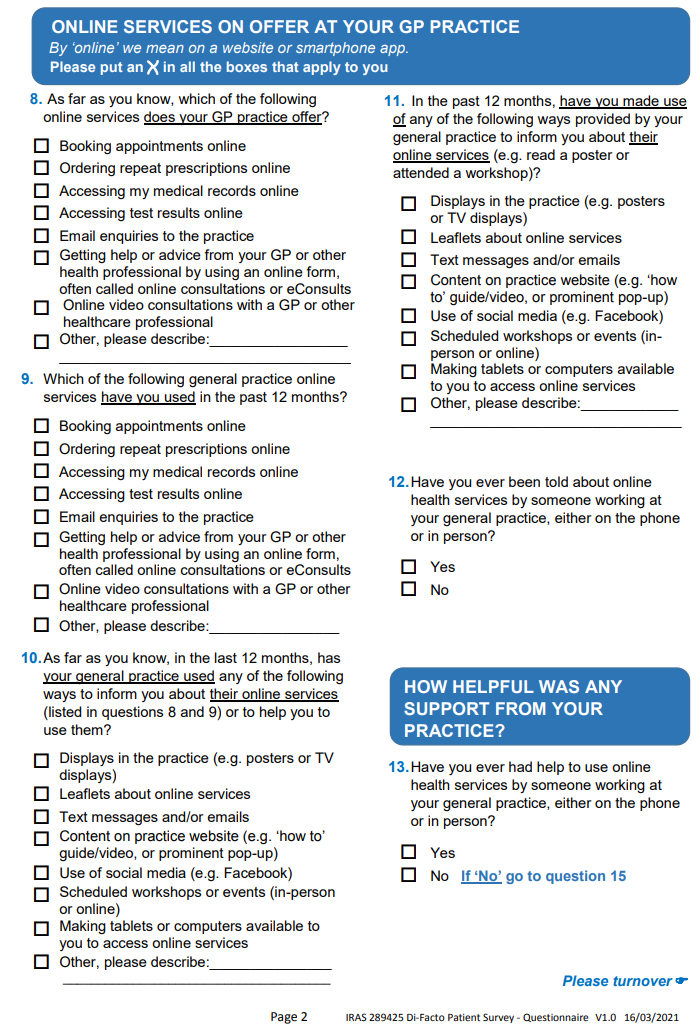


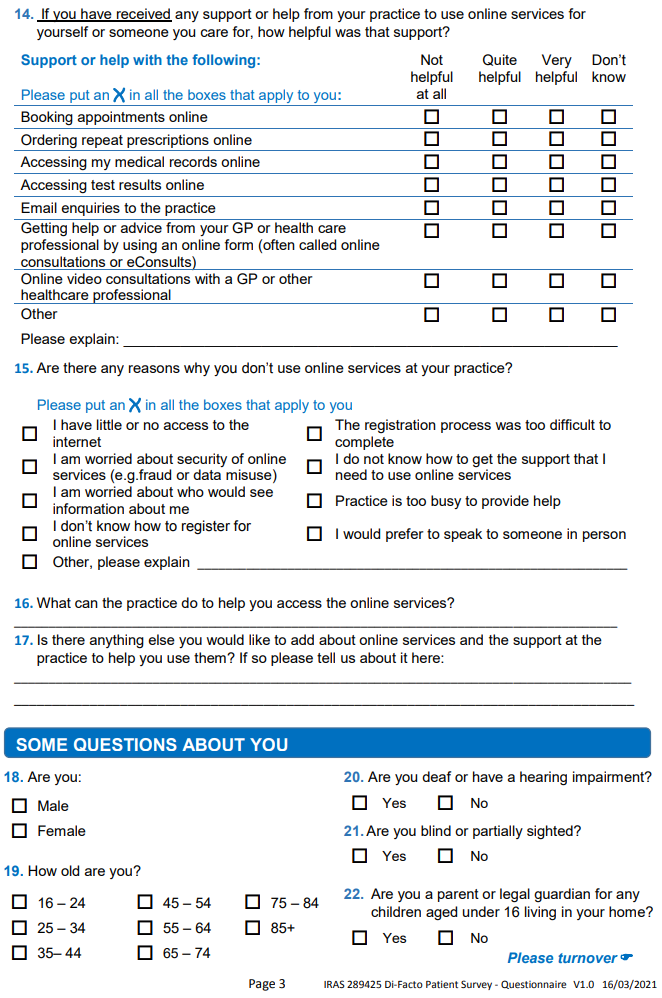


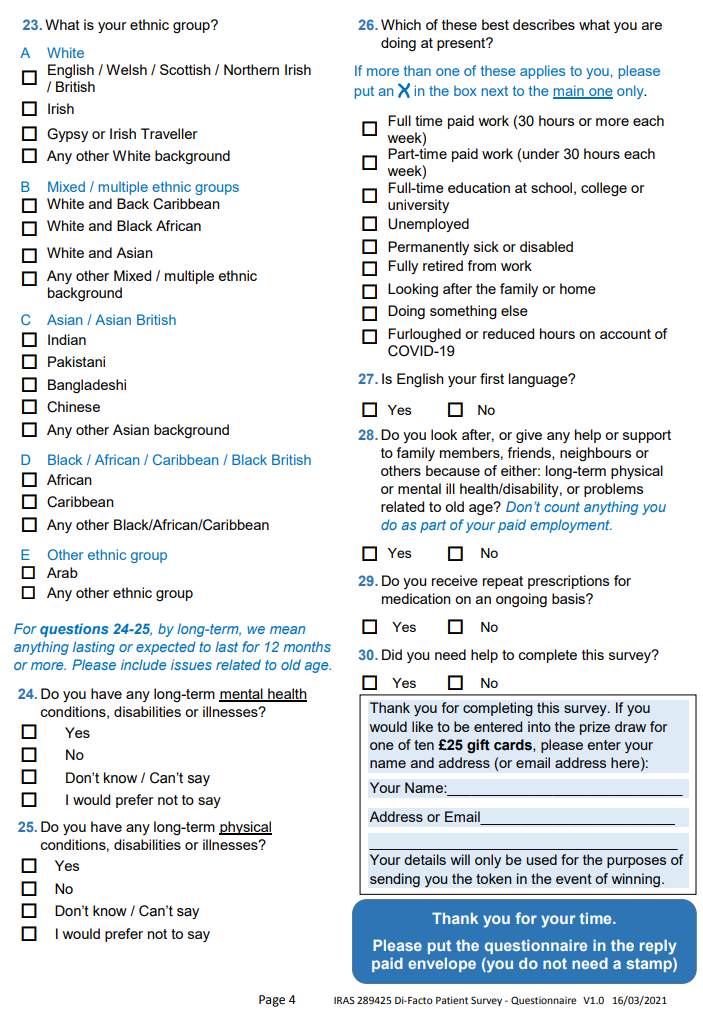

Supplement: Multimedia Appendix 2 [file jmir_v26i1e56528_app2.docx]

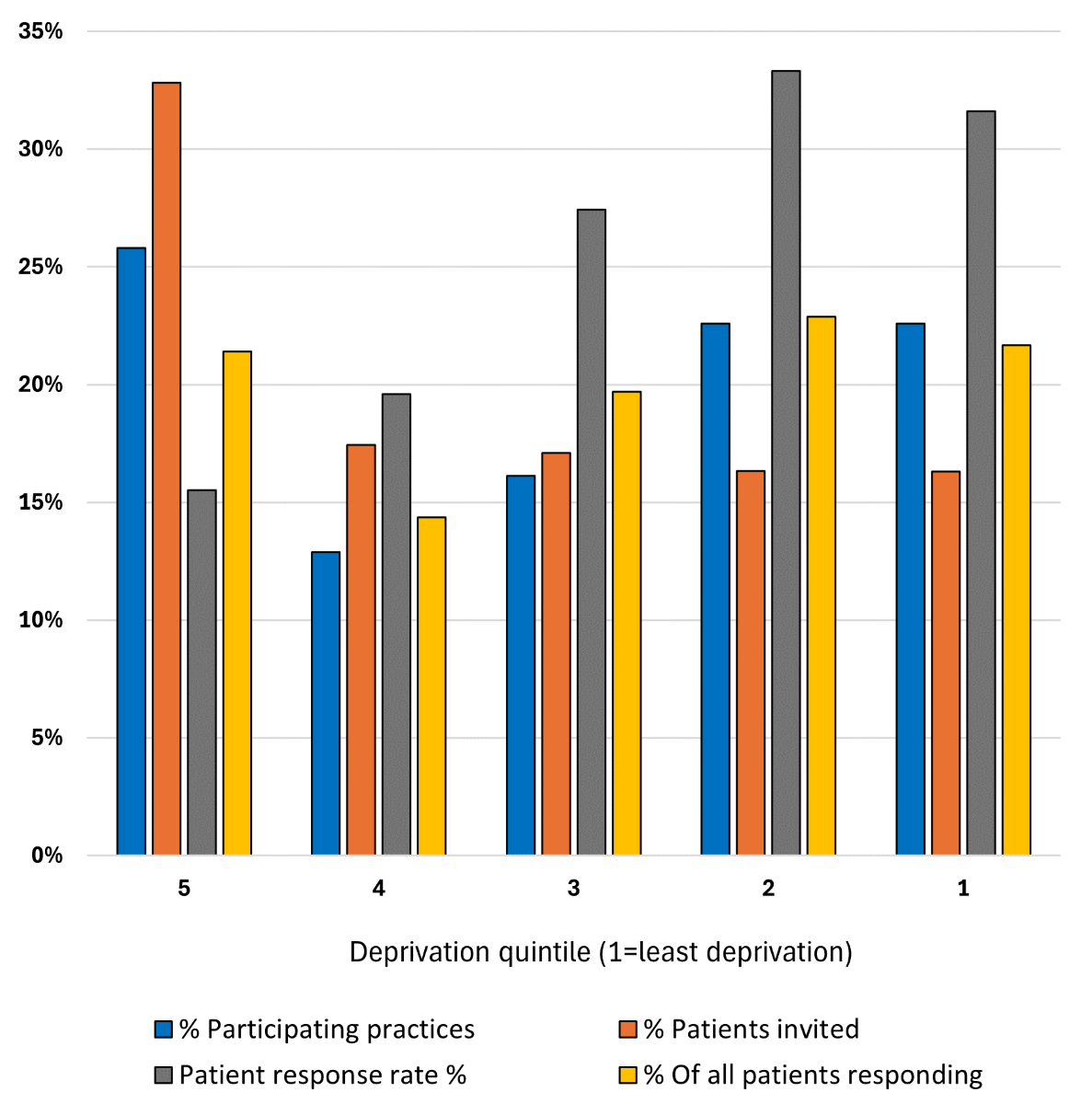

Supplement: Multimedia Appendix 14 [file jmir_v26i1e56528_app14.png]
