# Supplementary material for: Exploring How Patients Are Supported to Use Online Services in Primary Care in England Through “Digital Facilitation”: Survey Study
Source: J Med Internet Res. 2024 Aug 7;26:e56528. doi: 10.2196/56528 (PMC11339568; doi:10.2196/56528)
Supplement: Multimedia Appendix 3 [file jmir_v26i1e56528_app3.docx]

| Questions 2 to 6 of the patient survey focused on confidence in digital skills. For each question, a response of “Not very confident/ I can’t do this” scored zero, “Quite confident” scored one and “Very confident” scored two. Scores for the 5 questions were summed giving a score between 0 and 10. This was further split into three categories of 0-3 (not confident), 4-7 (quite confident) and 8-10 (very confident) |
| --- |
