# Supplementary material for: Exploring How Patients Are Supported to Use Online Services in Primary Care in England Through “Digital Facilitation”: Survey Study
Source: J Med Internet Res. 2024 Aug 7;26:e56528. doi: 10.2196/56528 (PMC11339568; doi:10.2196/56528)
Supplement: Multimedia Appendix 4 [file jmir_v26i1e56528_app4.docx]

|  |  | **Survey practices** | **Other practices in England** |
| --- | --- | --- | --- |
| **Deprivation quintile: according to patient postcode N (%)** | | | |
|  | 1 (least deprived) | 40 (25.6) | 1341 (19.89) |
|  | 2 | 43 (27.6) | 1337 (19.82) |
|  | 3 | 28 (17.9) | 1352 (20.05) |
|  | 4 | 24 (15.4) | 1356 (20.1) |
|  | 5 (most deprived | 21 (13.5) | 1359 (20.15) |
|  | Total | 155 (100) | 6745 (100) |
| **Rurality Urban and rural practices N (%)** | | | |
|  | Urban | 110 (70.5) | 5839 (85.33) |
|  | Rural | 46 (29.5) | 1004 (14.67) |
|  | Total | 156 (100) | 6843 (100) |
| **Age: Patients aged over 65 years (%)** | | | |
|  | Practices (N) | 155 | 6425 |
|  | Median | 20.3% | 17.7% |
|  | Mean | 20.1% | 17.6% |
|  | Std. dev. | 7.3% | 7.2% |
|  | Range | 4.5% to 41.7% | 0% to 98.5% |
| **List size: Practices with low, medium or high list sizes N (%)** | | | |
|  | ≤6,000 patients | 36 (23.2) | 2130 (33) |
|  | >6,000 & ≤12,000 | 65 (41.9) | 2854 (44.2) |
|  | >12,000 | 54 (34.8) | 1477 (22.8) |
|  | Total | 155 (100) | 6461 (100) |
| **Ethnicity: Patients of an ethnic minority in practices in England N (%)** | | | |
|  | Practices (n) | 155 | 6468 |
|  | Median | 4.8% | 7.5% |
|  | Mean | 12.5% | 17.0% |
|  | Std. dev. | 15.7% | 19.8% |
|  | Range | 1.0 to 86.8% | 0.0 to 90.5% |
|  |  |  |  |
