# Supplementary material for: Exploring How Patients Are Supported to Use Online Services in Primary Care in England Through “Digital Facilitation”: Survey Study
Source: J Med Internet Res. 2024 Aug 7;26:e56528. doi: 10.2196/56528 (PMC11339568; doi:10.2196/56528)
Supplement: Multimedia Appendix 5 [file jmir_v26i1e56528_app5.docx]

| *‘Which of the following* ***online*** *services do you* ***currently*** *offer to your patients (and their carers), and which services did you offer* ***before*** *the COVID-19 pandemic?’* | | | | | | |
| --- | --- | --- | --- | --- | --- | --- |
|  | **Offered currently**  **n (%)** | **Offered**  **pre-Covid**  **n (%)** | **Offered currently and pre-Covid**  **n (%)** | **Offered currently but not pre-Covid**  **n (%)** | **Offered**  **pre-Covid but not currently**  **n (%)** | **Never offered**  **n (%)** |
| **Online appointment booking (n=153)** | 78 (50.98) | 133 (86.93) | 66 (43.14) | 12 (7.84) | 67 (43.79) | 8 (5.23) |
| **Online repeat prescriptions (n=154)** | 143 (92.86) | 112 (72.73) | 101 (65.58) | 42 (27.27) | 11 (7.14) | 0 (0.00) |
| **Online access to medical records (n=151)** | 130 (86.09) | 99 (65.56) | 89 (58.94) | 41 (27.15) | 10 (6.62) | 11 (7.28) |
| **Test results (n=149)** | 114 (76.51) | 87 (58.39) | 77 (51.68) | 37 (24.83) | 10 (6.71) | 25 (16.78) |
| **Email enquiries (n=154)** | 126 (81.82) | 96 (62.34) | 83 (53.90) | 43 (27.92) | 13 (8.44) | 15 (9.74) |
| **Online consultations (n=151)** | 123 (81.46) | 69 (45.70) | 58 (38.41) | 65 (43.05) | 11 (7.28) | 17 (11.92) |
| **Video consultations (n=154)** | 143 (92.86) | 14 (9.09) | 11 (7.14) | 132 (85.71) | 3 (1.95) | 8 (5.84) |
| **Self-care resources (n=150)** | 126 (84.00) | 78 (52.00) | 68 (45.33) | 58 (38.67) | 10 (6.67) | 14 (9.33) |
| **Other (n=13)** | 11 (84.62) | 4 (30.77) | 3 (23.08) | 8 (61.54) | 1 (7.69) | 1 (7.69) |
