# Supplementary material for: Exploring How Patients Are Supported to Use Online Services in Primary Care in England Through “Digital Facilitation”: Survey Study
Source: J Med Internet Res. 2024 Aug 7;26:e56528. doi: 10.2196/56528 (PMC11339568; doi:10.2196/56528)
Supplement: Multimedia Appendix 6 [file jmir_v26i1e56528_app6.docx]

| *‘Which of the following activities have you carried out in your practice to either* ***promote*** *the use of the online primary care services*^a^ *listed above, or to* ***help or support patients*** *to use them?’*  ^a^ Provision of tablet/computers, workshops or events, practice champion and ad hoc support were all categorised a ‘active’ facilitation efforts, whereas all others were categorised as ‘passive’ | | | | | | |
| --- | --- | --- | --- | --- | --- | --- |
|  | **Promote**  **n (%)** | **Support**  **n (%)** | **Promote and support**  **n (%)** | **Promote but not support**  **n (%)** | **Support but not promote**  **n (%)** | **Neither**  **n (%)** |
| **Display (n=147)** | 122 (82.99) | 67 (45.58) | 57 (38.78) | 65 (44.22) | 10 (6.80) | 15 (10.20) |
| **Leaflet (n=141)** | 89 (63.12) | 57 (40.43) | 46 (32.62) | 43 (30.50) | 11 (7.80) | 41 (29.08) |
| **Text messages or emails (n=141)** | 110 (78.01) | 62 (43.97) | 48 (34.04) | 62 (43.97) | 14 (9.93) | 17 (12.06) |
| **Practice website (n=141)** | 125 (82.78) | 79 (52.32) | 64 (42.38) | 61 (40.40) | 15 (9.93) | 11 (7.28) |
| **Social media (n=148)** | 78 (52.70) | 45 (30.41) | 38 (25.68) | 40 (27.03) | 7 (4.73) | 63 (42.57) |
| **Ad hoc (n=143)** | 111 (77.62) | 87 (60.84) | 68 (47.55) | 43 (30.07) | 19 (13.29) | 13 (9.09) |
| **Practice champion (n=139)** | 30 (21.58) | 32 (23.02) | 16 (11.51) | 14 (10.07) | 16 (11.51) | 93 (66.91) |
| **Workshops or events (n=145)** | 11 (7.59) | 11 (7.59) | 4 (2.76) | 7 (4.83) | 7 (4.83) | 127 (87.59) |
| **Provision of tablets or computers (n=145)** | 10 (6.90) | 14 (9.66) | 7 (4.83) | 3 (2.07) | 7 (4.83) | 129 (88.97) |
| **Other (n=14)** | 1 (7.14) | 2 (14.29) | 1 (7.14) | 0 (0.00) | 1 (7.14) | 12 (85.71) |
