# Supplementary material for: Exploring How Patients Are Supported to Use Online Services in Primary Care in England Through “Digital Facilitation”: Survey Study
Source: J Med Internet Res. 2024 Aug 7;26:e56528. doi: 10.2196/56528 (PMC11339568; doi:10.2196/56528)
Supplement: Multimedia Appendix 7 [file jmir_v26i1e56528_app7.docx]

| *‘When you have promoted or supported activities (as in Q2), which services was it for?’* | | | | | | |
| --- | --- | --- | --- | --- | --- | --- |
|  | **Promote**  **n (%)** | **Support**  **n (%)** | **Promote and support**  **n (%)** | **Promote but not support**  **n (%)** | **Support but not promote**  **n (%)** | **Neither**  **n (%)** |
| **Online appointment booking (n=144)** | 106 (73.61) | 70 (48.61) | 53 (36.81) | 53 (36.81) | 17 (11.81) | 22 (15.28) |
| **Online repeat prescriptions (n=144)** | 118 (81.94) | 77 (53.47) | 56 (38.89) | 62 (43.06) | 21 (14.58) | 5 (3.47) |
| **Online access to medical records (n=144)** | 83 (59.29) | 57 (40.71) | 34 (24.29) | 49 (35.00) | 23 (16.43) | 34 (24.29) |
| **Test results (n=138)** | 83 (60.14) | 54 (39.13) | 31 (22.46) | 52 (37.68) | 23 (16.67) | 33 (23.91) |
| **Email enquiries (n=142)** | 73 (51.41) | 55 (38.73) | 32 (22.54) | 41 (28.87) | 23 (16.20) | 46 (32.39) |
| **eConsult (n=139)** | 98 (70.50) | 64 (46.04) | 48 (34.53) | 50 (35.97) | 16 (11.51) | 25 (17.99) |
| **Video consultations (n=142)** | 68 (47.89) | 69 (48.59) | 33 (23.24) | 35 (24.65) | 36 (25.35) | 38 (26.76) |
| **Self-care resources (n=142)** | 94 (66.20) | 69 (48.59) | 43 (30.28) | 51 (35.92) | 26 (18.31) | 22 (15.49) |
| **Other (n=12)** | 2 (16.67) | 3 (25.00) | 1 (8.33) | 1 (8.33) | 2 (16.67) | 8 (66.67) |
