# Supplementary material for: Exploring How Patients Are Supported to Use Online Services in Primary Care in England Through “Digital Facilitation”: Survey Study
Source: J Med Internet Res. 2024 Aug 7;26:e56528. doi: 10.2196/56528 (PMC11339568; doi:10.2196/56528)
Supplement: Multimedia Appendix 8 [file jmir_v26i1e56528_app8.docx]

| *‘When you have promoted or supported services which staff roles were involved?’* | |
| --- | --- |
| **Staff role** | **n (%)** |
| Doctors | 96 (61.54) |
| Nurses | 83 (53.21) |
| Other healthcare professionals | 65 (41.67) |
| Reception staff | 134 (85.90) |
| Administrative staff | 134 (85.90) |
| IT staff | 44 (28.21) |
| External contractors | 6 (3.85) |
| Staff specific duties to promote and support use of online services | 16 (10.26) |
| Volunteer | 31 (19.87) |
| Other | 6 (3.85) |
