# Supplementary material for: Exploring How Patients Are Supported to Use Online Services in Primary Care in England Through “Digital Facilitation”: Survey Study
Source: J Med Internet Res. 2024 Aug 7;26:e56528. doi: 10.2196/56528 (PMC11339568; doi:10.2196/56528)
Supplement: Multimedia Appendix 9 [file jmir_v26i1e56528_app9.docx]

| *‘When considering the promotion and support activities listed above^a^, which of the following groups of people (if any) do you specifically target?’*  ^a^ Provision of tablets or computers, workshops or events, practice champion and ad hoc support were all categorised a ‘active’ facilitation efforts, whereas all others were categorised as ‘passive’ | | | | |
| --- | --- | --- | --- | --- |
| **Patient group** | **N (%)** |  | **Patient group** | **N (%)** |
| Older adults | 87 (55.8) |  | People from minority ethnic communities | 45 (28.9) |
| People with physical health conditions | 68 (43.6) |  | People with limited or no internet access | 45 (28.9) |
| Patients with caring responsibilities or patient carers | 67 (43.0) |  | Lower income populations | 38 (24.4) |
| People with mental health conditions | 64 (41.0) |  | People with low literacy levels | 37 (23.7) |
| Socially isolated individuals | 53 (34.0) |  | People living in rural communities | 35 (22.4) |
| People with limited computer skills | 50 (32.1) |  | Other | 22 (14.1) |
| Non English speakers or those for whom English is a second language | 49 (31.4) |  |  |  |
