# Supplementary material for: Exploring How Patients Are Supported to Use Online Services in Primary Care in England Through “Digital Facilitation”: Survey Study
Source: J Med Internet Res. 2024 Aug 7;26:e56528. doi: 10.2196/56528 (PMC11339568; doi:10.2196/56528)
Supplement: Multimedia Appendix 10 [file jmir_v26i1e56528_app10.docx]

| ***’Following the national lockdown*** *starting on the 23^rd^ March 2020, has your* ***practice removed or reduced*** *access to any of the following offline services?’* | | | |
| --- | --- | --- | --- |
|  | **Access remained unchanged**  **n (%)** | **Access was reduced**  **n (%)** | **Access was removed**  **n (%)** |
| **Booking appointments in person (n=146)** | 23 (15.75) | 51 (34.93) | 72 (49.32) |
| **Booking appointments on the phone (n=146)** | 131 (89.73) | 14 (9.59) | 1 (0.69) |
| **Face to face consultations with a GP at the practice (n=146)** | 19 (13.01) | 115 (78.77) | 12 (8.22) |
| **Face to face consultations with a nurse at the practice (n=147)** | 48 (32.65) | 98 (66.67) | 1 (0.68) |
| **Provision of paper prescriptions (n=146)** | 40 (27.40) | 77 (52.74) | 29 (19.86) |
| **Ordering repeat prescriptions in person (n=147)** | 40 (27.21) | 69 (46.94) | 38 (25.85) |
| **Ordering repeat prescriptions by phone (n=135)** | 97 (71.85) | 20 (14.82) | 18 (13.33) |
| **Other (n=13)** | 13 (100.00) | 0 (0.00) | 0 (0.00) |
| *‘****Prior to the national lockdown*** *starting on the 23^rd^ March 2020, had your practice removed or reduced access to any of the following offline services, either* ***as a result of, or to encourage increasing use of online services?’*** | | | |
|  | **Access remained unchanged**  **n (%)** | **Access was reduced**  **n (%)** | **Access was removed**  **n (%)** |
| **Booking appointments in person (n=144)** | 103 (71.53) | 23 (15.97) | 18 (12.50) |
| **Booking appointments on the phone (n=145)** | 135 (93.10) | 9 (6.21) | 1 (0.69) |
| **Face to face consultations with a GP at the practice (n=145)** | 102 (70.34) | 39 (26.90) | 4 (2.76) |
| **Face to face consultations with a nurse at the practice (n=145)** | 119 (82.07) | 25 (17.24) | 1 (0.69) |
| **Provision of paper prescriptions (n=145)** | 80 (55.17) | 56 (38.62) | 9 (6.21) |
| **Ordering repeat prescriptions in person (n=144)** | 96 (66.67) | 35 (24.31) | 13 (9.03) |
| **Ordering repeat prescriptions by phone (n=141)** | 78 (55.32) | 34 (24.11) | 29 (20.57) |
| **Other (n=9)** | 6 (66.67) | 1 (11.11) | 2 (22.22) |
