# Supplementary material for: Exploring How Patients Are Supported to Use Online Services in Primary Care in England Through “Digital Facilitation”: Survey Study
Source: J Med Internet Res. 2024 Aug 7;26:e56528. doi: 10.2196/56528 (PMC11339568; doi:10.2196/56528)
Supplement: Multimedia Appendix 11 [file jmir_v26i1e56528_app11.docx]

| *’Please rate to what extent you agree or disagree with the following statements:’* | | | | | |
| --- | --- | --- | --- | --- | --- |
|  | **Strongly Agree**  **n (%)** | **Agree**  **n (%)** | **Neither agree nor disagree**  **n (%)** | **Disagree**  **n (%)** | **Strongly Disagree**  **n (%)** |
| **Responsibilities and impact of the Covid-19 pandemic:** |  |  |  |  |  |
| It is a general practice’s responsibility to inform patients what online primary care services are being offered (n=144) | 52 (36.11) | 71 (49.31) | 14 (9.72) | 7 (4.86) | 0 (0.00) |
| It is a general practice’s responsibility to support patients to use online primary care services (n=144) | 29 (20.14) | 76 (52.78) | 21 (14.58) | 12 (8.33) | 6 (4.17) |
| Other parts of the NHS (e.g. CCGs, NHS England) are responsible for informing patients about what online primary care services are being offered (n=144) | 41 (28.47) | 81 (56.25) | 18 (12.50) | 4 (2.78) | 0 (0.00) |
| Other parts of the NHS (e.g. CCGs, NHS England) are responsible for supporting patients to use online primary care services (n=143) | 38 (26.57) | 80 (55.94) | 21 (14.69) | 4 (2.80) | 0 (0.00) |
| COVID-19 has been a key driver in the uptake of online primary care services by patients (n=144) | 82 (56.94) | 47 (32.64) | 13 (9.03) | 2 (1.39) | 0 (0.00) |
| COVID-19 has inhibited our ability to support patients in using online primary care services (n=141) | 6 (4.26) | 27 (19.15) | 45 (31.91) | 52 (36.88) | 11 (7.80) |
| COVID-19 has led to an increase in the support we provide to patients in using online primary care services (n=144) | 32 (22.22) | 77 (53.47) | 28 (19.44) | 7 (4.86) | 0 (0.00) |
| **Demand, need, benefits and capacity to support patients to use online services:** | | | | | |
| The support we provide to patients to use online primary care services is in response to patient demand/need (n=142) | 25 (17.61) | 94 (66.20) | 18 (12.68) | 5 (3.52) | 0 (0.00) |
| The support we provide to patients to use online primary care services is in response to demand from practice staff (n=142) | 9 (6.34) | 52 (36.62) | 54 (38.03) | 22 (15.49) | 5 (3.52) |
| The support we provide to patients to use online primary care services is in response to demand from the CCG (n=142) | 8 (5.63) | 39 (27.46) | 60 (42.25) | 31 (21.83) | 4 (2.82) |
| Supporting patients to use online primary care services is a benefit to the practice (n=144) | 53 (36.81) | 73 (50.69) | 14 (9.72) | 4 (2.78) | 0 (0.00) |
| Supporting patients to use online primary care services is a benefit to the patients (n=144) | 62 (43.06) | 70 (48.61) | 10 (6.94) | 1 (0.69) | 1 (0.69) |
| We do not have the capacity to support patients in using online primary care services as much as we would like (n=141) | 34 (24.11) | 51 (36.17) | 31 (21.99) | 20 (14.18) | 5 (3.55) |
| **Level of support for patients, uptake, finance considerations and the future of online primary care services** | | | | | |
| Some patients are unlikely to use online primary care services no matter how much we support them (n=143) | 70 (48.95) | 65 (45.45) | 5 (3.50) | 3 (2.10) | 0 (0.00) |
| Some patient groups (e.g. older or more deprived patients) require more support in using online primary care services than others (n=143) | 59 (41.26) | 72 (50.35) | 12 (8.39) | 0 (0.00) | 0 (0.00) |
| We hope to increase uptake of online primary care services by supporting our patients to use them (n=143) | 27 (18.88) | 77 (53.85) | 33 (23.08) | 6 (4.20) | 0 (0.00) |
| We have managed to increase uptake of online primary care services by supporting our patients to use them (n=139) | 31 (22.30) | 78 (56.12) | 26 (18.71) | 3 (2.16) | 1 (0.72) |
| Financial incentives received by the practice influenced our decision to increase the support we provide to patients to use online primary care (n=135) | 5 (3.70) | 15 (11.11) | 53 (39.26) | 43 (31.85) | 19 (14.07) |
| Increased patient uptake of online primary care services leads to operational efficiencies for the practice (n=142) | 29 (20.42) | 66 (46.48) | 33 (23.24) | 11 (7.75) | 3 (2.11) |
| Online primary care services are complementary to traditional forms of access (n=143) | 26 (18.18) | 95 (66.43) | 14 (9.79) | 6 (4.20) | 2 (1.40) |
| Online primary care services will ultimately replace traditional forms of access (n=142) | 11 (7.75) | 41 (28.87) | 31 (21.83) | 46 (32.39) | 13 (9.15) |
