# Supplementary material for: Exploring How Patients Are Supported to Use Online Services in Primary Care in England Through “Digital Facilitation”: Survey Study
Source: J Med Internet Res. 2024 Aug 7;26:e56528. doi: 10.2196/56528 (PMC11339568; doi:10.2196/56528)
Supplement: Multimedia Appendix 12 [file jmir_v26i1e56528_app12.docx]

| *‘Are you aware of, and have you used either the Royal College of General Practitioners Patient Online toolkit, or the NHS England online consultations in primary care toolkit?* | | | | | | |
| --- | --- | --- | --- | --- | --- | --- |
|  | **Aware of the toolkit**  **n (%)** | **Have used the toolkit**  **n (%)** | **Aware and used**  **n (%)** | **Aware but not used**  **n (%)** | **Not aware but used**  **n (%)** | **Not aware**  **and not used**  **n (%)** |
| RCGP Patient Online toolkit (n=136) | 66 (48.53) | 13 (9.56) | 8 (5.88) | 58 (42.65) | 5(3.68) | 50 (36.76) |
| NHSE online consultations in primary care toolkit (n=137) | 55 (41.04) | 12 (8.96) | 6 (4.48) | 49 (36.57) | 6 (4.48) | 57 (42.54) |
