# Supplementary material for: Exploring How Patients Are Supported to Use Online Services in Primary Care in England Through “Digital Facilitation”: Survey Study
Source: J Med Internet Res. 2024 Aug 7;26:e56528. doi: 10.2196/56528 (PMC11339568; doi:10.2196/56528)
Supplement: Multimedia Appendix 13 [file jmir_v26i1e56528_app13.docx]

|  |  | **Main patient survey** |
| --- | --- | --- |
| Practice invitation | Invited | 179 |
|  | Declined | 31 |
| Practice response | Interest expressed | 74 |
|  | Agreement signed | 68 |
| Patient mail out | Completed | 62 |
|  | Patients invited | 12,822 |
| Patient response | Paper | 2448 |
|  | Online | 603 |
|  | Total | 3,051 (23.8%) |
