# Supplementary material for: Exploring How Patients Are Supported to Use Online Services in Primary Care in England Through “Digital Facilitation”: Survey Study
Source: J Med Internet Res. 2024 Aug 7;26:e56528. doi: 10.2196/56528 (PMC11339568; doi:10.2196/56528)
Supplement: Multimedia Appendix 15 [file jmir_v26i1e56528_app15.docx]

| **Digital confidence score** | **Total patient (%)**  **(N = 3051)** |
| --- | --- |
| Very confident (score 8-10) | 1589 (53.7) |
| Quite confident (score 4-7) | 704 (23.8) |
| Not confident (score 0-3) | 668 (22.6) |
