# Supplementary material for: Exploring How Patients Are Supported to Use Online Services in Primary Care in England Through “Digital Facilitation”: Survey Study
Source: J Med Internet Res. 2024 Aug 7;26:e56528. doi: 10.2196/56528 (PMC11339568; doi:10.2196/56528)
Supplement: Multimedia Appendix 16 [file jmir_v26i1e56528_app16.docx]

|  | **Awareness of any facilitation efforts^a^** | | **Use of any facilitation efforts^b^** | | **Being told about online services** | | **Being helped to use online services** | |
| --- | --- | --- | --- | --- | --- | --- | --- | --- |
|  | **Unadjusted OR (95% CI)** | ***P* value** | **Unadjusted OR (95% CI)** | ***P* value** | **Unadjusted OR (95% CI)** | ***P* value** | **Unadjusted OR (95% CI)** | ***P* value** |
|  | ^a^Awareness of any facilitation efforts includes respondents who ticked any of the first seven options of Q10.  ^b^Use of any facilitation efforts includes respondents who ticked any of the first seven options of Q11. | | | | | | | |
| **Model 1: Unadjusted** | | | | | | | | |
| Digital Confidence Measure |  |  |  |  |  |  |  |  |
| Very confident | Ref |  |  |  |  |  |  |  |
| Quite confident | 0.84 (0.70,1.02) | <.001 | 1.02 (0.85,1.22) | <.001 | 0.84 (0.69,1.03) | <.001 | 0.86 (0.66,1.11) | <.001 |
| Not confident | 0.31 (0.26, 0.38) |  | 0.40 (0.32, 0.49) |  | 0.51 (0.41, 0.64) |  | 0.32 (0.22, 0.46) |  |
| Gender |  |  |  |  |  |  |  |  |
| Male | Ref |  |  |  |  |  |  |  |
| Female | 0.93 (0.80,1.08) | .337 | 0.95 (0.82,1.11) | .542 | 1.24 (1.06,1.47) | .009 | 0.96 (0.77,1.19) | .710 |
| Age |  |  |  |  |  |  |  |  |
| 16-24 | 0.82 (0.56,1.21) | <.001 | 0.71 (0.48,1.05) | <.001 | 1.32 (0.89,1.96) | <.001 | 1.11 (0.65,1.88) | .005 |
| 25-34 | 1.22 (0.86,1.72) |  | 1.20 (0.86,1.68) |  | 1.27 (0.90,1.80) |  | 1.05 (0.65,1.69) |  |
| 35-44 | 1.08 (0.79,1.46) |  | 1.02 (0.76,1.38) |  | 1.25 (0.91,1.71) |  | 1.35 (0.91, 2.02) |  |
| 45-54 | 1.03 (0.79,1.33) |  | 1.01 (0.78,1.30) |  | 1.13 (0.86,1.48) |  | 1.34 (0.95,1.90) |  |
| 55-64 | Ref |  |  |  |  |  |  |  |
| 65-74 | 0.72 (0.58, 0.90) |  | 0.75 (0.60, 0.94) |  | 0.84 (0.66,1.07) |  | 0.91 (0.65,1.26) |  |
| 75-84 | 0.44 (0.34, 0.56) |  | 0.57 (0.44, 0.73) |  | 0.70 (0.53, 0.93) |  | 0.72 (0.49,1.05) |  |
| ≥ 85 | 0.35 (0.24, 0.53) |  | 0.32 (0.20, 0.51) |  | 0.39 (0.23, 0.66) |  | 0.37 (0.17, 0.83) |  |
| Deaf or hearing impairment |  |  |  |  |  |  |  |  |
| No | Ref |  |  |  |  |  |  |  |
| Yes | 0.70 (0.55, 0.87) | .002 | 0.85 (0.67,1.08) | .183 | 0.64 (0.49, 0.84) | .001 | 1.00 (0.71,1.39) | .985 |
| Parent |  |  |  |  |  |  |  |  |
| No | Ref |  |  |  |  |  |  |  |
| Yes | 1.59 (1.28,1.99) | <.001 | 1.32 (1.06,1.63) | .012 | 1.41 (1.13,1.77) | .003 | 1.43 (1.07,1.91) | .015 |
| Ethnicity |  |  |  |  |  |  |  |  |
| White | Ref |  |  |  |  |  |  |  |
| Other | 1.71 (1.24, 2.36) | .001 | 1.91 (1.41, 2.59) | <.001 | 1.15 (0.84,1.59) | .386 | 2.20 (1.54,3.14) | <.001 |
|  |  |  |  |  |  |  |  |  |
| **Model 1: Unadjusted** | | | | | | | | |
| Longterm physical or mental health condition | |  |  |  |  |  |  |  |
| No | Ref |  |  |  |  |  |  |  |
| Yes | 1.04 (0.90,1.22) | .573 | 1.09 (0.93,1.27) | .282 | 1.15 (0.97,1.35) | .105 | 1.28 (1.02,1.60) | .033 |
| Working status |  |  |  |  |  |  |  |  |
| Work | Ref |  |  |  |  |  |  |  |
| Education | 0.62 (0.38,1.02) | <.001 | 0.70 (0.43,1.15) | <.001 | 1.23 (0.75, 2.02) | <.001 | 1.10 (0.57, 2.11) | .101 |
| Other | 0.75 (0.58, 0.97) |  | 0.90 (0.69,1.16) |  | 0.93 (0.71,1.23) |  | 1.26 (0.89,1.78) |  |
| Retired | 0.55 (0.47, 0.65) |  | 0.61 (0.52, 0.72) |  | 0.70 (0.58, 0.84) |  | 0.82 (0.64,1.05) |  |
| First language |  |  |  |  |  |  |  |  |
| English | Ref |  |  |  |  |  |  |  |
| Other | 1.64 (1.23, 2.19) | .001 | 2.07 (1.57,2.72) | <.001 | 1.44 (1.08,1.92) | .013 | 2.00 (1.43, 2.81) | <.001 |
| Repeat prescription |  |  |  |  |  |  |  |  |
| No | Ref |  |  |  |  |  |  |  |
| Yes | 0.97 (0.83,1.14) | 0.737 | 1.16 (0.98,1.36) | .079 | 1.29 (1.09,1.54) | .004 | 1.24 (0.98,1.57) | .072 |

| **Model 2: Adjusted** |  |  |  |  |  |  |  |  |
| --- | --- | --- | --- | --- | --- | --- | --- | --- |
| Digital Confidence Measure |  |  |  |  |  |  |  |  |
| Very confident | Ref |  |  |  |  |  |  |  |
| Quite confident | 0.85 (0.69,1.06) | <.001 | 1.04 (0.84, 1.28) | <.001 | 0.87 (0.69, 1.09) | .005 | 0.93 (0.69, 1.25) | <.001 |
| Not confident | 0.35 (0.27,0.46) |  | 0.39 (0.29, 0.51) |  | 0.61 (0.46, 0.83) |  | 0.32 (0.20, 0.51) |  |
| Gender |  |  |  |  |  |  |  |  |
| Male | Ref |  |  |  |  |  |  |  |
| Female | 0.95 (0.80,1.13) | .581 | 0.92 (0.78, 1.09) | .333 | 1.14 (0.95, 1.37) | .147 | 0.86 (0.67, 1.10) | .220 |

|  | **Awareness of any facilitation efforts^a^** | | **Use of any facilitation efforts^b^** | | **Being told about online services** | | **Being helped to use online services** | |
| --- | --- | --- | --- | --- | --- | --- | --- | --- |
|  | **Unadjusted OR (95% CI)** | ***P*-value** | **Unadjusted OR (95% CI)** | ***P*-value** | **Unadjusted OR (95% CI)** | ***P*-value** | **Unadjusted OR (95% CI)** | ***P*-value** |
|  | ^a^Awareness of any facilitation efforts includes respondents who ticked any of the first seven options of Q10.  ^b^Use of any facilitation efforts includes respondents who ticked any of the first seven options of Q11. | | | | | | |  |
| **Model 2: Adjusted** | | | | | | | | |

| Age |  |  |  |  |  |  |  |  |
| --- | --- | --- | --- | --- | --- | --- | --- | --- |
| 16-24 | 0.75 (0.44,1.27) | .181 | 0.60 (0.35, 1.03) | .159 | 1.51 (0.88, 2.58) | .478 | 0.93 (0.43, 2.03) | .176 |
| 25-34 | 0.94 (0.64,1.40) |  | 0.99 (0.68, 1.45) |  | 1.06 (0.71, 1.59) |  | 1.06 (0.61, 1.84) |  |
| 35-44 | 0.91(0.62,1.33) |  | 0.97 (0.66, 1.41) |  | 1.05 (0.71, 1.57) |  | 1.37 (0.82, 2.30) |  |
| 45-54 | 0.88 (0.65,1.19) |  | 0.92 (0.68, 1.23) |  | 1.01 (0.73, 1.38) |  | 1.30 (0.86, 1.98) |  |
| 55-64 | Ref |  |  |  |  |  |  |  |
| 65-74 | 0.82 (0.61,1.11) |  | 1.00 (0.74, 1.34) |  | 0.83 (0.61, 1.15) |  | 0.90 (0.58, 1.38) |  |
| 75-84 | 0.60 (0.42,0.86) |  | 0.83 (0.58, 1.19) |  | 0.79 (0.54, 1.16) |  | 0.69 (0.40, 1.17) |  |
| ≥ 85 | 0.59 (0.34,1.02) |  | 0.48 (0.26, 0.90) |  | 0.52 (0.27, 1.01) |  | 0.15 (0.03, 0.67) |  |
| Deaf or hearing impairment | | | | | | | | |
| No | Ref |  |  |  |  |  |  |  |
| Yes | 1.00 (0.75,1.33) | .994 | 1.24 (0.93, 1.66) | .146 | 0.86 (0.62, 1.18) | .352 | 1.45 (0.95, 2.20) | .084 |
| Parent |  |  |  |  |  |  |  |  |
| No | Ref |  |  |  |  |  |  |  |
| Yes | 1.19 (0.88,1.59) | .255 | 0.98 (0.73, 1.30) | .875 | 1.22 (0.90, 1.65) | .204 | 0.99 (0.67, 1.47) | .967 |
| Ethnicity |  |  |  |  |  |  |  |  |
| White | Ref |  |  |  |  |  |  |  |
| Other | 1.49 (0.99, 2.22) | .053 | 1.50 (1.03, 2.18) | .035 | 0.95 (0.64, 1.41) | .793 | 1.83 (1.15, 2.91) | .011 |
| Longterm physical or mental health condition | | | | | | | | |
| No | Ref |  |  |  |  |  |  |  |
| Yes | 1.26 (1.04,1.53) | .017 | 1.18 (0.98, 1.43) | .089 | 1.07 (0.87, 1.31) | .526 | 1.30 (0.98, 1.71) | .068 |
| Working status | | | | | | | | |
| Work | Ref |  |  |  |  |  |  |  |
| Education | 0.74 (0.38, 1.44) | .301 | 1.06 (0.54, 2.07) | .514 | 1.11 (0.57, 2.18) | .972 | 1.26 (0.50, 3.16) | .271 |
| Other | 0.78 (0.58, 1.05) |  | 0.85 (0.64, 1.15) |  | 0.97 (0.72, 1.33) |  | 1.40 (0.94, 2.08) |  |
| Retired | 1.02 (0.77, 1.36) |  | 0.83 (0.62, 1.10) |  | 1.04 (0.77, 1.41) |  | 1.37 (0.90, 2.08) |  |
|  |  |  |  |  |  |  |  |  |

|  | **Awareness of any facilitation efforts^a^** | | **Use of any facilitation efforts^b^** | | **Being told about online services** | | **Being helped to use online services** | |
| --- | --- | --- | --- | --- | --- | --- | --- | --- |
|  | **Unadjusted OR (95% CI)** | ***P*-value** | **Unadjusted OR (95% CI)** | ***P*-value** | **Unadjusted OR (95% CI)** | ***P*-value** | **Unadjusted OR (95% CI)** | ***P*-value** |
| **Model 2: Adjusted** | | | | | | | | |

| First language |  |  |  |  |  |  |  |  |
| --- | --- | --- | --- | --- | --- | --- | --- | --- |
| English | Ref |  |  |  |  |  |  |  |
| Other | 1.56 (1.08, 2.26) | .017 | 2.00 (1.41, 2.84) | <.001 | 1.62 (1.12, 2.34) | .011 | 1.71 (1.09, 2.70) | .021 |
| Repeat prescription | | | | | | | | |
| No | Ref |  |  |  |  |  |  |  |
| Yes | 1.28 (1.04, 1.57) | .022 | 1.46 (1.18, 1.80) | <.001 | 1.71 (1.36, 2.14) | <.001 | 1.46 (1.07, 2.00) | .016 |
