# Supplementary material for: Exploring How Patients Are Supported to Use Online Services in Primary Care in England Through “Digital Facilitation”: Survey Study
Source: J Med Internet Res. 2024 Aug 7;26:e56528. doi: 10.2196/56528 (PMC11339568; doi:10.2196/56528)
Supplement: Multimedia Appendix 17 [file jmir_v26i1e56528_app17.docx]

|  | **Awareness of any facilitation efforts^a^** | | **Use of any facilitation efforts^b^** | | **Being told about online services** | | **Being helped to use online services** | |
| --- | --- | --- | --- | --- | --- | --- | --- | --- |
|  | **Adjusted OR**  **(95% CI)** | ***P*-value** | **Adjusted OR**  **(95% CI)** | ***P*-value** | **Adjusted OR**  **(95% CI)** | ***P*-value** | **Adjusted OR**  **(95% CI)** | ***P*-value** |
|  | **^a^**Awareness of any facilitation efforts includes respondents who ticked any of the first seven options of Q10.  **^b^** Use of any facilitation efforts includes respondents who ticked any of the first seven options of Q11. | | | | | | | |
| **Adjusted Model 1** | | | | | | | | |
| Gender |  |  |  |  |  |  |  |  |
| Male | Ref |  |  |  |  |  |  |  |
| Female | 0.90 (0.76, 1.07) | 0.221 | 0.89 (0.75, 1.05) | 0.175 | 1.12 (0.93, 1.34) | 0.236 | 0.87 (0.68, 1.11) | 0.258 |
| Age |  |  |  |  |  |  |  |  |
| 16-24 | 0.87 (0.51, 1.47) | <0.001 | 0.65 (0.38, 1.12) | <0.001 | 1.63 (0.95, 2.81) | 0.105 | 1.01 (0.47, 2.21) | 0.015 |
| 25-34 | 1.06 (0.72, 1.58) |  | 1.06 (0.73, 1.56) |  | 1.13 (0.76, 1.70) |  | 0.97 (0.55, 1.71) |  |
| 35-44 | 0.91 (0.62, 1.34) |  | 0.98 (0.67, 1.44) |  | 1.08 (0.72, 1.62) |  | 1.40 (0.83, 2.36) |  |
| 45-54 | 0.92 (0.68, 1.25) |  | 0.95 (0.70, 1.28) |  | 1.03 (0.75, 1.42) |  | 1.30 (0.86, 1.98) |  |
| 55-64 | Ref |  |  |  |  |  |  |  |
| 65-74 | 0.70 (0.52, 0.95) |  | 0.91 (0.68, 1.22) |  | 0.80 (0.58, 1.10) |  | 0.87 (0.57, 1.35) |  |
| 75-84 | 0.42 (0.30, 0.59) |  | 0.63 (0.44, 0.89) |  | 0.68 (0.46, 0.99) |  | 0.52 (0.31, 0.89) |  |
| ≥ 85 | 0.34 (0.20, 0.60) |  | 0.29 (0.16, 0.55) |  | 0.42 (0.21, 0.83) |  | 0.11 (0.02, 0.47) |  |
| Deaf/hearing impairment | |  |  |  |  |  |  |  |
| No | Ref |  |  |  |  |  |  |  |
| Yes | 1.09 (0.81, 1.45) | 0.583 | 1.30 (0.97, 1.75) | 0.079 | 0.81 (0.58, 1.13) | 0.210 | 1.36 (0.89, 2.08) | 0.153 |
| Parent |  |  |  |  |  |  |  |  |
| No | Ref |  |  |  |  |  |  |  |
| Yes | 1.27 (0.94, 1.71) | 0.120 | 1.01 (0.75, 1.35) | 0.954 | 1.29 (0.95, 1.75) | 0.107 | 1.05 (0.70, 1.57) | 0.816 |
| Ethnicity |  |  |  |  |  |  |  |  |
| White | Ref |  |  |  |  |  |  |  |
| Other | 1.48 (0.97, 2.25) | 0.070 | 1.46 (0.99, 2.16) | 0.057 | 1.01 (0.67, 1.53) | 0.946 | 1.97 (1.23, 3.15) | 0.005 |
| Long-term physical or mental health condition | | |  |  |  |  |  |  |
| No | Ref |  |  |  |  |  |  |  |
| Yes | 1.25 (1.04, 1.52) | 0.020 | 1.18 (0.98, 1.43) | 0.081 | 1.10 (0.90, 1.35) | 0.363 | 1.27 (0.97, 1.68) | 0.087 |
|  |  |  |  |  |  |  |  |  |
|  |  |  |  |  |  |  |  |  |
|  |  |  |  |  |  |  |  |  |
| **Adjusted Model 1** |  |  |  |  |  |  |  |  |
| Working status |  |  |  |  |  |  |  |  |
| Work | Ref |  |  |  |  |  |  |  |
| Education | 0.75 (0.38, 1.48) | 0.670 | 1.06 (0.54, 2.09) | 0.446 | 1.04 (0.53, 2.06) | 0.919 | 1.32 (0.52, 3.34) | 0.368 |
| Other | 0.86 (0.63, 1.16) |  | 0.89 (0.66, 1.20) |  | 1.12 (0.82, 1.53) |  | 1.37 (0.92, 2.05) |  |
| Retired | 0.97 (0.73, 1.29) |  | 0.80 (0.60, 1.06) |  | 1.02 (0.75, 1.38) |  | 1.28 (0.84, 1.95) |  |
| First language |  |  |  |  |  |  |  |  |
| English | Ref |  |  |  |  |  |  |  |
| Other | 1.38 (0.94, 2.02) | 0.103 | 1.81 (1.25, 2.61) | 0.002 | 1.60 (1.08, 2.36) | 0.018 | 1.80 (1.13, 2.87) | 0.013 |
| Repeat prescription |  |  |  |  |  |  |  |  |
| No | Ref |  |  |  |  |  |  |  |
| Yes | 1.28 (1.04, 1.58) | 0.019 | 1.47 (1.19, 1.81) | <0.001 | 1.71 (1.36,2.14) | <0.001 | 1.49 (1.09, 2.03) | 0.013 |

| **Adjusted Model 2** | | | | | | | | |
| --- | --- | --- | --- | --- | --- | --- | --- | --- |
| Digital Confidence Measure | |  |  |  |  |  |  |  |
| Very confident | Ref |  |  |  |  |  |  |  |
| Quite confident | 0.84 (0.68, 1.05) | <0.001 | 1.05 (0.85, 1.31) | <0.001 | 0.86 (0.68, 1.09) | 0.031 | 0.97 (0.72, 1.32) | <0.001 |
| Not confident | 0.40 (0.30, 0.53) |  | 0.44 (0.33, 0.60) |  | 0.66 (0.48, 0.90) |  | 0.33 (0.20, 0.55) |  |
| Gender |  |  |  |  |  |  |  |  |
| Male | Ref |  |  |  |  |  |  |  |
| Female | 0.94 (0.79, 1.11) | 0.457 | 0.91 (0.76, 1.08) | 0.280 | 1.13 (0.93, 1.36) | 0.215 | 0.86 (0.67, 1.11) | 0.242 |
| Age |  |  |  |  |  |  |  |  |
| 16-24 | 0.78 (0.46, 1.34) | 0.163 | 0.63 (0.36, 1.09) | 0.189 | 1.56 (0.90, 2.70) | 0.574 | 0.97 (0.44, 2.13) | 0.207 |
| 25-34 | 0.95 (0.64, 1.43) |  | 1.01 (0.68, 1.49) |  | 1.07 (0.71, 1.62) |  | 0.95 (0.53, 1.69) |  |
| 35-44 | 0.87 (0.59, 1.29) |  | 0.97 (0.66, 1.42) |  | 1.08 (0.72, 1.62) |  | 1.41 (0.83, 2.39) |  |
| 45-54 | 0.88 (0.64, 1.19) |  | 0.94 (0.70, 1.28) |  | 1.03 (0.75, 1.42) |  | 1.33 (0.87, 2.04) |  |
| 55-64 | Ref |  |  |  |  |  |  |  |
| 65-74 | 0.80 (0.59, 1.08) |  | 1.01 (0.75, 1.36) |  | 0.85 (0.62, 1.18) |  | 0.95 (0.62, 1.48) |  |
| 75-84 | 0.56 (0.39, 0.81) |  | 0.80 (0.55, 1.15) |  | 0.80 (0.54, 1.19) |  | 0.70 (0.41, 1.20) |  |
| ≥ 85 | 0.58 (0.32, 1.05) |  | 0.49 (0.25, 0.94) |  | 0.56 (0.28, 1.15) |  | 0.19 (0.04, 0.87) |  |

|  | **Awareness of any facilitation efforts^a^** | | **Use of any facilitation efforts^b^** | | **Being told about online services** | | **Being helped to use online services** | |
| --- | --- | --- | --- | --- | --- | --- | --- | --- |
|  | **Adjusted OR**  **(95% CI)** | ***P*-value** | **Adjusted OR**  **(95% CI)** | ***P*-value** | **Adjusted OR**  **(95% CI)** | ***P*-value** | **Adjusted OR**  **(95% CI)** | ***P*-value** |

| **Adjusted Model 2** | |  |  |  |  |  |  |  |
| --- | --- | --- | --- | --- | --- | --- | --- | --- |
| Deaf/hearing impairment | |  |  |  |  |  |  |  |
| No | Ref |  |  |  |  |  |  |  |
| Yes | 1.14 (0.84, 1.54) | 0.395 | 1.36 (1.00, 1.84) | 0.047 | 0.83 (0.59, 1.17) | 0.286 | 1.46 (0.95, 2.25) | 0.083 |
| Parent |  |  |  |  |  |  |  |  |
| No | Ref |  |  |  |  |  |  |  |
| Yes | 1.18 (0.87, 1.60) | 0.284 | 0.96 (0.72, 1.29) | 0.797 | 1.22 (0.90, 1.66) | 0.204 | 1.01 (0.68, 1.51) | 0.957 |
| Ethnicity |  |  |  |  |  |  |  |  |
| White | Ref |  |  |  |  |  |  |  |
| Other | 1.46 (0.95, 2.24) | 0.081 | 1.46 (0.98, 2.17) | 0.060 | 1.05 (0.69, 1.58) | 0.830 | 1.99 (1.24, 3.20) | 0.005 |
|  |  |  |  |  |  |  |  |  |
| Long-term physical or mental health condition | |  |  |  |  |  |  |  |
| No | Ref |  |  |  |  |  |  |  |
| Yes | 1.26 (1.04, 1.53) | 0.021 | 1.18 (0.97, 1.43) | 0.099 | 1.09 (0.89, 1.34) | 0.424 | 1.27 (0.96, 1.68) | 0.098 |
| Working status |  |  |  |  |  |  |  |  |
| Work | Ref |  |  |  |  |  |  |  |
| Education | 0.77 (0.39, 1.52) | 0.741 | 1.07 (0.54, 2.13) | 0.683 | 1.07 (0.54, 2.13) | 0.924 | 1.35 (0.53, 3.41) | 0.184 |
| Other | 0.89 (0.65, 1.21) |  | 0.91 (0.67, 1.24) |  | 1.11 (0.81, 1.53) |  | 1.47 (0.98, 2.21) |  |
| Retired | 1.03 (0.77, 1.37) |  | 0.84 (0.63, 1.12) |  | 1.05 (0.77, 1.43) |  | 1.40 (0.91, 2.13) |  |
| First language |  |  |  |  |  |  |  |  |
| English | Ref |  |  |  |  |  |  |  |
| Other | 1.46 (0.98, 2.17) | 0.060 | 1.89 (1.30, 2.76) | <0.001 | 1.54 (1.04, 2.29) | 0.032 | 1.89 (1.18, 3.02) | 0.008 |
| Repeat prescription |  |  |  |  |  |  |  |  |
| No | Ref |  |  |  |  |  |  |  |
| Yes | 1.28 (1.03, 1.58) | 0.024 | 1.46 (1.18, 1.80) | <0.001 | 1.71 (1.36, 2.15) | <0.001 | 1.49 (1.08, 2.04) | 0.014 |
| \| **^a^**Awareness of any facilitation efforts includes respondents who ticked any of the first seven options of Q10.  **^b^**Use of any facilitation efforts includes respondents who ticked any of the first seven options of Q11. \| \| --- \| | | | | | | | | |
