# Supplementary material for: Exploring How Patients Are Supported to Use Online Services in Primary Care in England Through “Digital Facilitation”: Survey Study
Source: J Med Internet Res. 2024 Aug 7;26:e56528. doi: 10.2196/56528 (PMC11339568; doi:10.2196/56528)
Supplement: Multimedia Appendix 18 [file jmir_v26i1e56528_app18.docx]

| **Mode of facilitation** | **% patients aware in practices using mode of facilitation** | **% patients aware in practices not using mode of facilitation** | ***P* value** | **% patients using in practices using mode of facilitation** | **% patients using in practices not using mode of facilitation** | ***P* value** |
| --- | --- | --- | --- | --- | --- | --- |
| Displays  (N = 2,935) | 17.55%  (466/2,655) | 10.71%  (30/280) | .004 | 9.64%  (256/2,655) | 7.50%  (21/280) | .244 |
| Leaflets  (N = 2,776) | 5.39%  (92/1,707) | 6.27%  (67/1,069) | .333 | 2.75%  (47/1,707) | 3.18%  (34/1,069) | .515 |
| SMS text or emails  (N = 2,828) | 39.14%  (1,068/2,729) | 43.43%  (43/99) | .390 | 28.22%  (770/2,729) | 25.25%  (25/99) | .519 |
| Web content  (N = 3,032) | 7.43%  (197/2,650) | 8.38%  (32/382) | .514 | 5.09%  (135/2,650) | 4.45%  (17/382) | .590 |
| Social media  (N = 2,975) | 6.06%  (105/1,733) | 0.97%  (12/1,242) | <.001 | 3.64%  (63/1,733) | 1.21%  (15/1,242) | <.001 |
| Workshops or events  (N = 2,874) | 1.50%  (4/267) | 0.46%  (12/2,607) | .030 | 1.50% (4/267) | 0.38%  (10/2,607) | .013 |
| Tablets or computers  (N = 2,921) | 0.65%  (2/306) | 0.54%  (14/2,615) | .791 | 0.98% (3/306) | 0.42%  (11/2,615) | .180 |
