# Supplementary material for: Exploring How Patients Are Supported to Use Online Services in Primary Care in England Through “Digital Facilitation”: Survey Study
Source: J Med Internet Res. 2024 Aug 7;26:e56528. doi: 10.2196/56528 (PMC11339568; doi:10.2196/56528)
Supplement: Multimedia Appendix 19 [file jmir_v26i1e56528_app19.docx]

| **Mode of facilitation** | **% (n/N) patients informed of any facilitation in practices using mode of facilitation** | **% (n/N) patients informed of any facilitation in practices not using mode of facilitation** | ***P* value** | **% (n/N) patients using any support in practices using mode of facilitation** | **% (n/N) patients using any support in practices not using mode of facilitation** | ***P* value** |
| --- | --- | --- | --- | --- | --- | --- |
| Ad hoc Promotion or  support by practice staff | 30.01%  (696/2,319) | 31.41%  (120/382) | .581 | 13.12%  (301/2,294) | 15.00%  (57/380) | .319 |
| Employ or train a ‘practice champion’ with specific responsibility in this area | 36.34%  (149/410) | 29.04%  (669/2,304) | .003 | 16.01%  (65/406) | 13.07%  (298/2,280) | .110 |
