# Supplementary material for: Exploring How Patients Are Supported to Use Online Services in Primary Care in England Through “Digital Facilitation”: Survey Study
Source: J Med Internet Res. 2024 Aug 7;26:e56528. doi: 10.2196/56528 (PMC11339568; doi:10.2196/56528)
Supplement: Multimedia Appendix 20 [file jmir_v26i1e56528_app20.docx]

| **MODE OF FACILITATION** | **% of patient group informed of any facilitation in practices targeting this group** | **% of patient group informed of any facilitation in practices not targeting this group** | ***P* (n/N) alue** | **% of patient group using any facilitation in practices targeting this group** | **% of patient group using any facilitation in practices not targeting this group** | ***P*  Value** |
| --- | --- | --- | --- | --- | --- | --- |
| Older adults (65 years or older) (N = 1,371) | 47.44%  (343/723) | 43.98%  (285/648) | .199 | 34.58%  (250/723) | 31.02%  (201/648) | .161 |
| People with physical health conditions (e.g. sight loss, mobility impairments, multi-morbidity (N = 1,044) | 52.35%  (256/489) | 55.32%  (307/555) | .338 | 38.04%  (186/489) | 41.98%  (233/555) | .194 |
| People with mental health conditions (e.g. depression, psychosis) (N = 470) | 59.32%  (105/177) | 50.85%  (149/293) | .074 | 40.11%  (71/177) | 41.98%  (123/293) | .690 |
| People with limited or no internet access (N = 279) | 25.56%  (23/90) | 22.75%  (43/189) | .606 | 13.33%  (12/90) | 18.52%  (35/189) | .279 |
| Non-English speakers or those for whom English is a second language (N = 246) | 59.55%  (53/89) | 65.61%  (103/157) | .343 | 48.31%  (43/89) | 59.24%  (93/157) | .098 |
| People from ethnic minority communities (N = 206) | 63.04%  (58/92) | 66.67%  (76/114) | .588 | 53.26%  (49/92) | 55.26%  (63/114) | .774 |
| Patients with caring responsibilities or patient carers (N = 696) | 52.11%  (148/284) | 54.61%  (225/412) | .516 | 38.73%  (110/284) | 39.32%  (162/412) | .876 |
